# A novel atypical sperm centriole is functional during human fertilization

**DOI:** 10.1038/s41467-018-04678-8

**Published:** 2018-06-07

**Authors:** Emily L. Fishman, Kyoung Jo, Quynh P. H. Nguyen, Dong Kong, Rachel Royfman, Anthony R. Cekic, Sushil Khanal, Ann L. Miller, Calvin Simerly, Gerald Schatten, Jadranka Loncarek, Vito Mennella, Tomer Avidor-Reiss

**Affiliations:** 1 Department of Biological Sciences, University of Toledo, 2801W. Bancroft, Toledo, OH 43607 USA; 2 Cell Biology Program, The Hospital for Sick Children, Department of Biochemistry, University of Toronto, 555 University Avenue, Toronto, ON M5G 1X8 Canada; 3 Laboratory of Protein Dynamics and Signaling, Center for Cancer Research, National Cancer Institute, 1050 Boyles Street, Frederick, MD 21702 USA; 4 Department of Molecular, Cellular, and Developmental Biology, University of Michigan, 830 North University Ave, Ann Arbor, MI 48109 USA; 5 Departments of Cell Biology; Obstetrics, Gynecology and Reproductive Sciences; and Bioengineering, Magee-Womens Research Institute, University of Pittsburgh School of Medicine, 204 Craft Avenue, Pittsburgh, PA 15213 USA

## Abstract

The inheritance of the centrosome during human fertilization remains mysterious. Here we show that the sperm centrosome contains, in addition to the known typical barrel-shaped centriole (the proximal centriole, PC), a surrounding matrix (pericentriolar material, PCM), and an atypical centriole (distal centriole, DC) composed of splayed microtubules surrounding previously undescribed rods of centriole luminal proteins. The sperm centrosome is remodeled by both reduction and enrichment of specific proteins and the formation of these rods during spermatogenesis. In vivo and in vitro investigations show that the flagellum-attached, atypical DC is capable of recruiting PCM, forming a daughter centriole, and localizing to the spindle pole during mitosis. Altogether, we show that the DC is compositionally and structurally remodeled into an atypical centriole, which functions as the zygote’s second centriole. These findings now provide novel avenues for diagnostics and therapeutic strategies for male infertility, and insights into early embryo developmental defects.

## Abstract (web summary)

The two zygote centrioles are paternally inherited; however, their development is incompletely understood. Here, the authors show that the distal centriole is remodeled into an atypical centriole which functions as the zygote’s second centriole.

## Introduction

Human development begins with the zygote, which divides many times to produce all of the somatic cells. These somatic cells each contain two centrioles, which duplicate in a number-controlled manner from pre-existing centrioles. Since cells require two centrioles for normal division, one would expect the human zygote to have two centrioles during interphase and four centrioles during mitosis. However, four centrioles have never been shown in any mammalian zygotes; only three centrioles were observed^1,;2^. Since the human oocyte lacks centrioles, and the zygote’s paternal pronucleus is associated with an aster of microtubules, it seems that the embryo’s centrioles are paternally inherited^3,4^. The paternal centrioles reside at the junction of the sperm nucleus and flagellum, in a region known as the neck. The neck region includes, in addition to the centrioles, the striated columns, and capitulum, which surround an electron-light region known as the vault^5,6^.

**Fig. 1:**
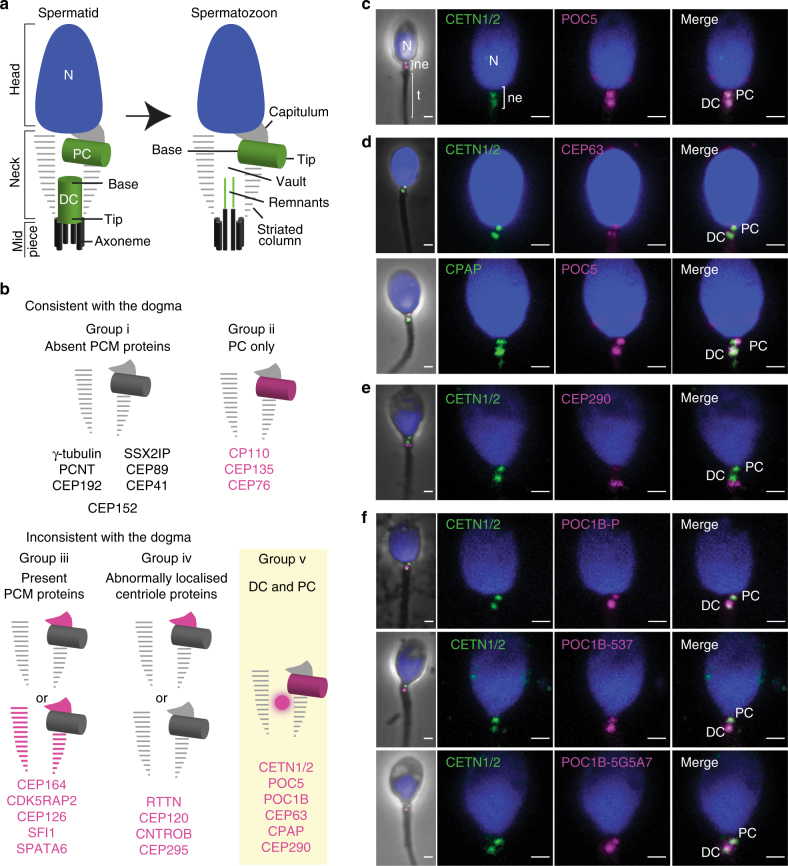
Centriolar proteins localize to the DC (group v). **a** The current dogma is that mature sperm have one centriole. The spermatid neck has a PC and DC, and the ejaculated spermatozoon has a PC and an empty space, the vault, which marks the location of the degenerated DC (Manandhar et al.^6^). **b** Graphical summary of the groups of the localization patterns of proteins in the sperm. Group i is centriolar proteins that were localized to the PC area only and is consistent with the dogma. Group ii is PCM proteins that were absent from the sperm, as is consistent with the dogma. Group iii are PCM proteins that were unexpectedly localized to the striated columns or capitulum. Group iv are centriolar proteins that were unexpectedly localized to the striated columns or capitulum, or were unexpectedly absent altogether. Group v are proteins that were unexpectedly present in the DC and the PC, which are inconsistent with the dogma. Group v is shown in **c**–**f**. **c** Antibodies against the centriole tip proteins CETN1/2 and POC5 labeled the PC and DC. **d** Antibodies against CEP63 and CPAP labeled near the CETN1/2 or POC5-labeled PC and DC. **e** Antibodies against CEP290 labeled adjacent to the CETN1/2-labeled DC, presumably marking the junction of the DC with the axoneme. **f** Three distinct antibodies against POC1B labeled both the PC and DC. Unlike other centriolar proteins, POC1B was enriched in the DC relative to the PC (POC1B-P: 2.1 ± 0.5, POC1B-537: 2.74 ± 1.64, POC1B-5G5A7: 1.92 ± 0.41, *N* > 6). POC1B-P, Rabbit polyclonal antibody from the Pearson lab; POC1B-537, Rabbit polyclonal antibody from the Avidor-Reiss lab; POC1B-5G5A7 Rat monoclonal Ab from the Avidor-Reiss lab. N nucleus, ne sperm neck, t tail; scale bars 1 μm

The current dogma is that the early sperm’s centrioles and their surrounding pericentriolar material (PCM) are modified by a process called centrosome reduction during spermiogenesis^5,7^. During reduction, the distal centriole’s (DC) typical structure disintegrates, the proteins surrounding the centrioles that make up the PCM are eliminated, and a vault appears in the expected place of the DC^8^. Only residual microtubules and proteins are observed in the mature spermatozoa DC, and no function has been associated with the DC remnants or the vault^6^ (Fig. 1a). Therefore, the current prevailing dogma is that the sperm has a single functional centriole, the PC^9–13^, and therefore the zygote inherits only one centriole. Yet, the zygotic somehow provides four centrioles, two for each daughter cell. The inheritance of only a single centriole is problematic since centrioles form by duplication of pre-existing centrioles, which act as a platform for the formation of a single nascent daughter centriole. If the zygote inherits only one functional centriole, then the origin of the second interphase centriole is unknown.

A similar enigma existed previously in insects until it was shown that insect sperm possesses, in addition to the known centriole, a second atypical centriolar structure^14–16^. This atypical structure has been shown to be essential for normal fertility and embryo development^15,17^. The presence of an unexpected second centriolar structure in insects raises the possibility that humans possess a similar atypical structure that may be important during reproduction and development. Here we investigated human spermatozoa and bovine zygotes to determine if mammals have a functional atypical second centriole.

In this work, we examine several groups of centrosomal proteins, and find that a subset of them are present, unexpectedly in the DC of human spermatozoa. Using Correlative Light and Electron Microscopy as well as High Pressure Freezing, Freeze Substitution Electron Microscopy, we find that the DC is attached to the base of the axoneme, but that its microtubules splay outward, forming a novel, atypical, structure. Using Super-Resolution microscopy we find that the human and bovine DC’s subset of centriolar proteins are organized into rods. During spermatogenesis in bovine testes, we find that the DC’s rods appear during spermatid development. Next, we test the competency of the DC using an in vitro system and find that the human DC can recruit the PCM protein, γ-tubulin. Furthermore, we follow the DC of bovine sperm into the zygote and found that it recruits PCM, forms an aster, forms a new daughter centriole, and localizes to the spindle pole, all while maintaining its attachment to the axoneme. These findings discover a novel, atypical centriole in the sperm, which functions in the zygote.

## Results

### The DC contains a subset of centriolar proteins

The dogma of centrosome reduction and previously published literature states that PCM proteins are reduced based on the observations that γ-tubulin and PCNT are missing from the sperm centrosome^5^. Likewise, we found that the PCM proteins γ-tubulin and PCNT were eliminated from the neck region during spermatogenesis (Supplementary Fig. 1a). Only one protein thus far has been seen in the DC, CETN1/2; however, CETN1/2 was seen primarily in the PC, and only inconsistently in the DC, which was interpreted to be undergoing reduction^5,6^.

Contrary to this observation, we found that an antibody against the centriolar protein CETN1/2 equally labeled both the DC and PC in ejaculated human sperm (Supplementary Fig. 1a)^5^. Because of this unexpected observation of CETN1/2 in the DC, we sought to define the composition of the DC to determine whether it could be the second centriole of the spermatozoa. We identified candidate sperm centrosome proteins by comparing the spermatozoon proteome^18–21^ to the centrosome database^22^ (Supplementary Fig. 2, Supplementary Table 1). We examined the localization of candidates using immunostaining (Supplementary Fig. 1). Then, we validated the specificity of the antibodies in U2OS cells (Supplementary Fig. 3).

The putative centrosomal proteins were separated into five groups based on their localization in the spermatozoan neck region (Fig. 1b). Consistent with centrosome reduction, we found that several PCM/appendage/satellite proteins such as CEP152 and CEP192 were absent from the neck (Group i) (Supplementary Fig. 1b), and similarly some centriolar proteins such as CEP135 and CEP76 were seen only to associate with the intact PC (Group ii) (Supplementary Fig. 1c). Both of these groups support the idea that the some proteins are exclusively present in the PC and missing from the PCM the DC.

However, contrary to centrosome reduction, we observed several PCM/appendage proteins, such as CEP164 and CDK5RAP2, in the capitulum and striated columns (Group iii) (Supplementary Fig. 1d). These findings support structural studies showing that the capitulum and striated columns form in association with centriole microtubules, suggesting that the neck structures are a specialized form of PCM^8^. Also, contrary to centrosome reduction, we found some centriolar proteins such as CEP295 and CNTROB were absent from the PC and were either observed in the PCM or were undetectable, suggesting that the composition of the PC is also altered during centrosome reduction (Group iv) (Supplementary Fig. 1e).

Finally, several centriolar proteins labeled the DC (Group v). Of these proteins, we found that centriole distal lumen proteins CETN1/2 and POC5^23^ localized equally to both the DC and PC (Fig. 1c). The PCM protein CEP63 and the centriole/PCM protein CPAP localized at the PC and DC (Fig. 1d). The transition zone protein, CEP290^24^, localized to the junction between the DC and the axoneme, presumably marking the tip of the DC (Fig. 1e). Interestingly, we found that a centriolar protein, POC1B, which is enriched in the atypical centriole of *Drosophila* sperm^15^, was similarly enriched in the DC relative to the PC. Three distinct antibodies confirmed that POC1B was enriched about two-fold in the DC in comparison to the PC (Fig. 1f). Altogether, these observations suggest that the DC is not eliminated during spermatogenesis but, instead, is present with an altered composition.

### The DC is made of splayed microtubules

**Fig. 2:**
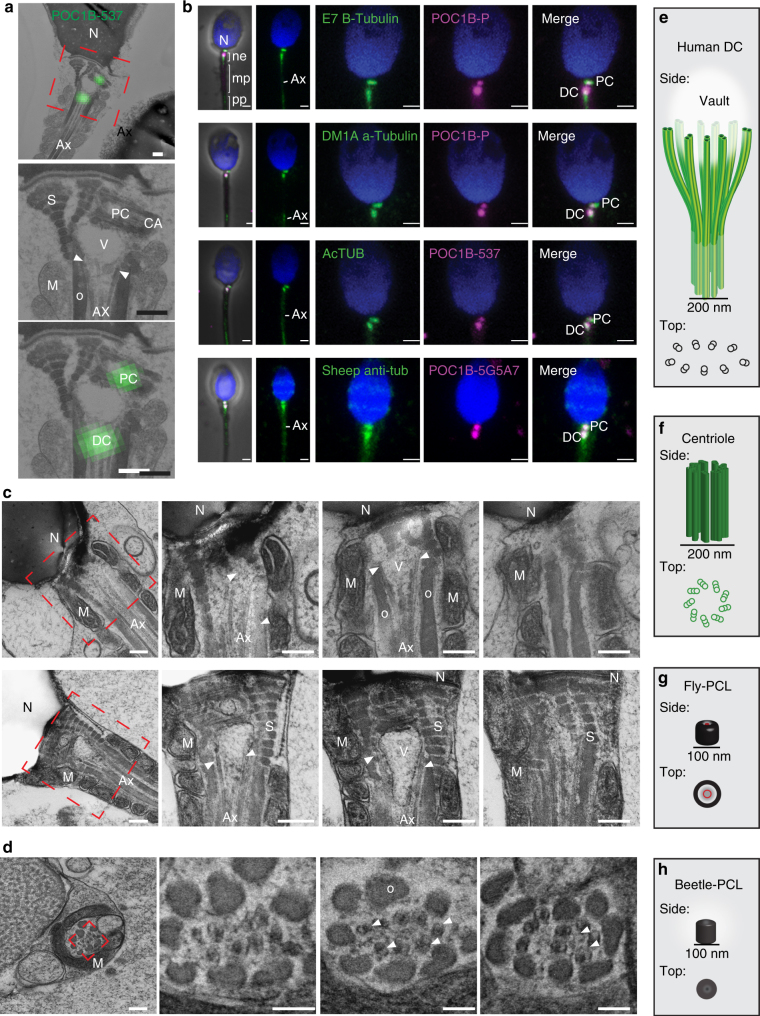
POC1B associates with splayed microtubules at the axoneme base. **a** The PC and the DC were labeled with POC1B using Correlative Light and Electron Microscopy. These structures were found to be associated with the splayed microtubules (arrowheads, between the axoneme and electron light vault). Scale bars 200 nm. **b** Four distinct antibodies against tubulins labeled the DC and the PC with greater intensity than in the axoneme. Scale bars 1 μm. **c**, **d** Two examples of serial longitudinal sections showed splayed microtubules around an electron light vault using high-pressure freezing-freeze substitution of the sperm neck (**c**). Serial cross-sections showed splayed microtubules (**d**, arrowheads). Scale bars 200 nm. **e**–**h** Models with side and top views of the human spermatozoon DC (**e**) and centriole (**f**), the fly spermatid's PCL (**g**), and beetle spermatid's PCL (**h**). Ax axoneme, M mitochondria, mp tail midpiece, N nucleus, ne neck, O outer dance fibers, pp tail principal piece, S striated columns, V vault

The POC1B enrichment of the DC was unexpected, and therefore we investigated the POC1B localization more precisely, using Correlative Light and Electron Microscopy. We found POC1B in the PC as well as at the base of the axoneme, which we interpret to be the DC (Fig. 2a). The DC is located between the vault and the axoneme, flanked by the outer dense fibers and the striated columns. This suggests that the DC is at the base of the axoneme, and the vault is located between the two centrioles.

To precisely identify the location of the DC’s microtubules, we studied tubulin and POC1B, using four anti-tubulin antibodies (anti-β-tubulin E7, anti-α-tubulin DM1A, anti-acetylated-tubulin, and sheep anti-tubulin). Each of the four anti-tubulin antibodies labeled two foci. Both of these foci colocalized with POC1B in the PC and the DC (Fig. 2b). In some cases, the tubulin signals in the PC and DC were more intense than in the midpiece axoneme, possibly because the PC and DC microtubules were unmasked during centrosome reduction. These findings suggest that the DC microtubules were not degenerated as was previously believed.

To determine the location of the DC’s microtubules, we used transmission electron microscopy (TEM) with high-pressure freezing-freeze substitution, which better preserves cellular structural integrity when compared to chemical fixation. We found that the DC microtubules splayed out from the axoneme, surrounding the vault, and extended up to the PC (Fig. 2c, d). The splayed microtubules extend from the cylindrical axoneme and open up forming an inverted cone that ends with an ovoid base facing the nucleus (Fig. 2e). The presence of these microtubules may have been overlooked previously because they were not in the typical nine-fold symmetric pattern (Fig. 2f) and they may have been disrupted by the classical chemical fixation methods used for TEM. Structurally atypical centrioles have been observed previously in insect sperm and are known as the proximal centriole-like structures (PCL)^15,17,25–27^. However, the atypical DC is distinct from that of the PCL in fly (Fig. 2g) or beetle (Fig. 2h), suggesting that sperm centrioles evolve diverse structures.

### The DC proteins are organized into rods

**Fig. 3:**
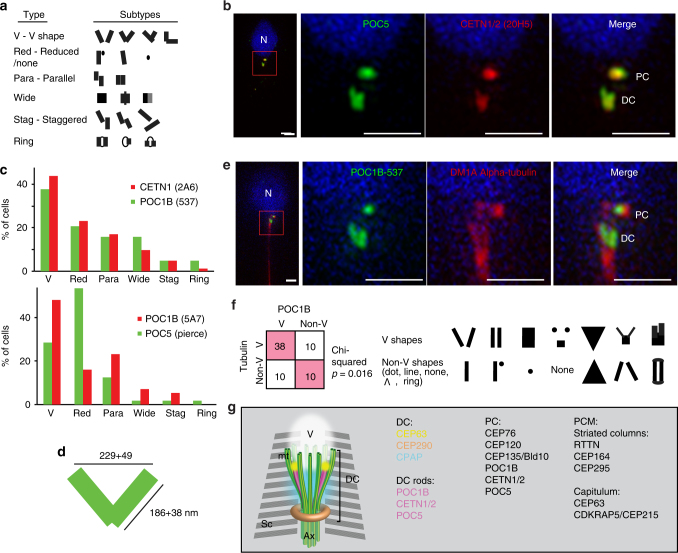
The precise location of DC proteins. **a** Diagrams of the six types of DC rods morphologies. **b** 3D-SIM showed “V”-shaped rods of centriolar proteins POC5 and CETN1/2. **c** Graph depicting the abundance of the six types of DC rods morphologies. The most common type for both CETN1/2 and POC1B is “V” shape (N~140). POC5 shows both a high rate of “V” shape (~25%), and a higher rate of “reduced” shape. **d** A diagram depicting the dimensions of the rods (height and width) and their distances from each other (from center to center of the rods) at their tips and bases (*N* ≥ 20). **e** 3D-SIM showed “V”-shaped rods of centriolar protein POC1B flanking the splayed microtubules shown with DM1A, α-tubulin (red). **f** Quantification of the relationship between POC1B shape and tubulin shape in 3D-SIM. Most cells that have “V”-shaped tubulin also have “V”-shaped POC1B, suggesting that their morphology correlates. *p* = 0.016 by Chi-squared. **g** Model of the DC in the ejaculated spermatozoon based on electron microscopy, confocal microscopy, and 3D-SIM. Sc striated columns, Ax axoneme, V vault. Scale bars 1 μm

To precisely localize the DC proteins, we performed sub-diffraction resolution microscopy. We found that CEP63, which normally forms a ring around the centriole, localized as two dots, and CPAP, which is commonly found in the centriole, localized around the DC (Supplementary Fig. 4a–b). Importantly, POC1B, CETN1/2, and POC5 exhibited rod-like distribution in various orientations (Supplementary Fig. 4c). We identified six types of orientations. The first, most common, was “V” shaped, where the rods were angled away from each other. The second was “Reduced” orientation, when one or both rods had a reduced size, appearing as a single rod, a rod and a dot, a dot, or no signal at all. Third was “Parallel”, where the rods were parallel. The fourth orientation we found was “Wide”, where presumably, the rods were close to each other. Fifth was “Staggered”, where the rods were in various orientations. And finally, the sixth orientation was “Ring”, where the rods appeared to be connected by thin lines (Fig. 3a). Four of these categories included rods in different orientations (“V”, “Parallel”, “Wide”, and “Staggered”), but the two others cannot be explained as a different view of the same configuration, suggesting that the relative position between the rods may be varied. However, in 40% of spermatozoa imaged, the rods appeared in a “V” shape suggesting this may be the preferred orientation (Fig. 3b, c). A similar “V” shape was also observed in STED (Supplementary Fig. 4d).

The length of the individual rods in the “V” is about 190 nm, which is about half the length of the distal lumen of a typical centriole (~350 nm)^28,29^ (Fig. 3d). The width of the “V” tip opening is about 230 nm, which is much wider than a typical centriole lumen (~130 nm)^28^. The “V” was in line with the axoneme and was flanked by the splayed-out DC microtubules (Fig. 3e, f). This suggests that the rods associate with the splayed microtubules of the DC.

Further examination of our electron microscopy images finds electron-dense material along the splaying DC microtubules that might be the structure underlying the rods (Supplementary Fig. 5). Similarly, Manandhar et al.^5^ found using immunogold labeling that CETN1/2 associates with microtubules at the point where the microtubules begin to splay in the DC, the same site where we observed the DC rods. This rod organization has never been described for any centriolar protein; most centriolar proteins and the structures within the distal lumen of a centriole exhibit radial symmetry in the form of disk and column structures^30–33^. Together, these findings demonstrate that several centrosomal proteins have an atypical distribution in the DC (Fig. 3g).

### Bovine DC undergo remodeling

To further study centrosome remodeling and DC function during sperm development, we examined bovine testes because bovine sperm, like human sperm, was thought to contain only the PC^12,34^. Like human spermatozoa, POC1B, POC5, CETN1/2, and tubulin labeled the DC of bovine spermatozoa (Supplementary Fig. 6a–b), and the spermatozoa lack CEP152 (an essential PCM protein) and SAS-6 (a daughter centriole marker) (Supplementary Fig. 6b–c). Furthermore, the CEP152, SAS-6, POC1B, POC5, and CETN1/2 antibodies recognize the centrosomes of undifferentiated bovine cells, validating the antibody specificity (Supplementary Fig. 6d–h). In addition, bovine spermatozoa shows an obvious “V” shape in the DC using HyVolution confocal microscopy, suggesting that the bovine DC is both similar in shape to, and bigger than the human DC (Supplementary Fig. 6i–j). Together, the compositional similarities and size increase make bovine sperm a good model to examine the finer detail of the DC and its development.

**Fig. 4:**
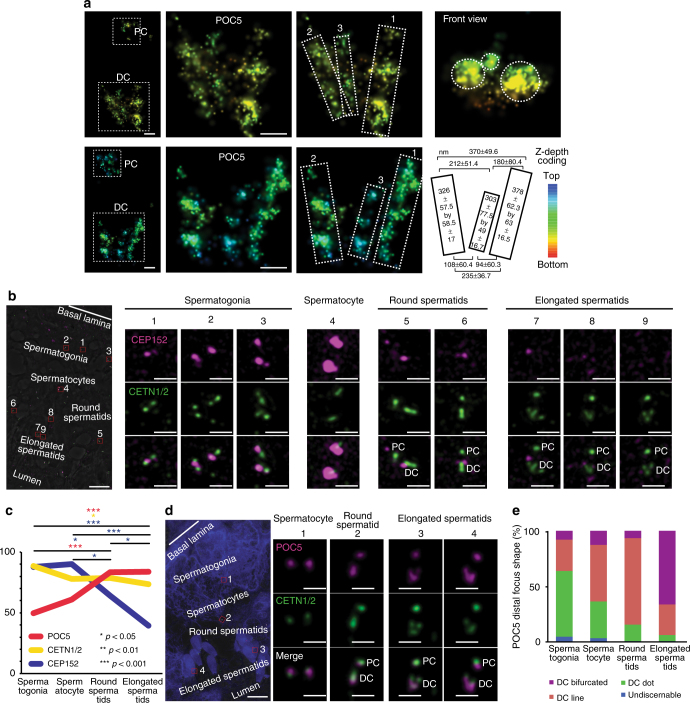
POC5 and CETN 1/2 are enriched and redistributed, while CEP152 is reduced. **a** STORM with POC5 antibodies recognized the PC and DC (left panel). Zoom in on the DC (two middle panels) identifies two major rods (marked as “1” and “2”) and one minor rod (marked as “3”). A diagram (second row, right panel) depicting the dimensions of the rods and their distances from each other (from center to center) at their tips and bases (*N* ≥ 7). Scale bar 100 nm. **b** A section of a seminiferous tubule (left panel) depicting the changes in CEP152 (pink) and CETN1/2 (green) in various stages (cells are numbered 1–9). During spermatogenesis, CEP152 and CENT1/2 localizes to 2–4 foci in spermatogonia (cells 1–3). In spermatocytes, CEP152 is maintained, often in large foci surrounding much smaller CENT1/2 lines (cell 4). In round spermatids, CEP152 is localized to the ends of elongated CENT1/2 foci and reduces into two small foci straddling the CENT1/2 line (cells 5–6). Finally, in the elongated spermatids (cells 7–9), CEP152 is localized to the tips of the CENT1/2 “V” shape, before it is finally dramatically reduced. Scale bar 10 μm in low-magnification images (left), and 1 μm in centriole high-magnification images (right). **c** During spermatogenesis, CEP152 is dramatically reduced (blue), CETN1/2 is slightly reduced (yellow), and POC5 is enriched (red) (*N* ≥ 3). *p*-values determined by ANOVA with Fisher’s LSD post hoc. **d** A section of a seminiferous tubule (left panel) depicting the changes in the distribution of POC5 (pink) and CETN1/2 (green) in various stages (cells 1–4). Both POC5 and CENT1/2 localize to dot-shaped centrioles in spermatocytes (cell 1). In round spermatids, both POC5 and CENT1/2 elongate into a DC that is longer than the PC (cell 2). Finally, in the elongated spermatids (cells 3–4) both CETN1/2 and POC5 appear as “V” shapes. Scale bar 10 μm in low-magnification images (left), and 1 μm in centriole high-magnification images (right). **e** Quantification of the DC shape by POC5 immunolabeling during spermatogenesis. Most spermatogonia are dot shaped; most round spermatids are elongated; and most elongated spermatids are bifurcated (*N* ≥ 3, *p* ≤ 0.00001). *p* by Chi-squared

The advantages of bovine sperm allowed us to closer examination of the DC rods using stochastic optical reconstruction microscopy (STORM), which has a resolution limit of ~30 nm. In a side-view projection, we found that the bovine DC is made of two main rods and one minor rod (Fig. 4a). Likewise, a 90° rotation projection clearly showed three dots representing a top-view of the rods. The two major rods are about 350 nm long, whereas the minor rod is only about 300 nm long. The length is twice as long as the human DC rods, which agrees with our findings using HyVolution confocal microscopy (Figs. 3d, 4a, Supplementary Fig. 6j). The distance between the tops of the rods is approximately 370 nm, which is substantially wider than a typical centriole. The distance between the rods at the base is only 230 nm, which is similar to the width of a typical centriole lumen. Together, these dimensions suggest that the base of the rod’s “V” shape is around the same size as a typical centriole lumen, but then opens up much wider than a typical centriole.

To gain insight into the process of DC remodeling, we tracked centrosomal proteins during bovine spermatogenesis. We found that while CEP152 is present in spermatogonia, spermatocytes, and round spermatids, its levels are dramatically reduced in elongated spermatids (Fig. 4b, c). In contrast, we observed that as CEP152 is dramatically reduced (by ~55%), CETN1/2 is slightly reduced (by ~17%), and POC5 is enriched (by ~68%) (Fig. 4c, d). A close look at POC5 localization revealed that POC5 and CETN1/2 transforms from a rounded focus, to an elongated focus, and then to “V” during the transition from round spermatids to elongated spermatids (Fig. 4b, d, e). These findings suggest that centriole remodeling is accompanied by both protein reduction and protein enrichment and that the structure of the DC is remodeled during spermiogenesis, before the sperm has gained mobility.

### The human DC is competent in vitro

**Fig. 5:**
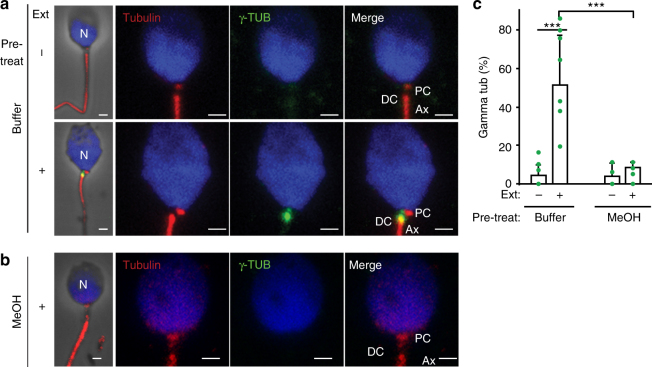
The remodeled DC is functional in vitro. **a**–**c** The human remodeled DC recruits γ-tubulin from *Xenopus* egg extract. Demembranated human ejaculated spermatozoa did not have γ-tubulin, but after exposure to the extract, γ-tubulin was present near the DC (**a**). Demembranated spermatozoa with a DC inactivated by methanol pretreatment did not recruit γ-tubulin (**b**). Quantification (*N* ≥ 3) of the percent of γ-tubulin-positive sperm pretreated with either buffer or methanol (MeOH) and then exposed to extract (+) or buffer (−) (**c**). *p* < 0.001 by *t*-test. Scale bar 1 μm. Error bars represent +1 standard deviation

To examine the function of the DC, we used a cell-free centrosome reconstitution system that exposes demembranated sperm to *Xenopus* egg extracts^34,35^. The exposed sperm centrioles can recruit PCM proteins typically involved in aster formation from the egg extract, thus simulating the fertilization environment without creating embryos. This system has previously demonstrated the essential role of the PCM protein γ-tubulin in centrosome assembly and function^35^. Later, using human sperm, it was found that the sperm centrosome was able to recruit γ-tubulin, but the specific site of recruitment was not investigated^34^. We employed this system to determine if the DC was capable of recruiting γ-tubulin and found that the main site of γ-tubulin recruitment was near the DC (Fig. 5a). This γ-tubulin recruitment activity was specific to the sperm, as sperm fixed with methanol were unable to recruit γ-tubulin (Fig. 5b, c). Altogether, this suggests that the remodeled DC is competent to recruit PCM.

### The bovine DC functions in zygote

Since the DC in human spermatozoa is competent to recruit PCM, it may function as the second centriole of the zygote. Indeed, in human zygotes, the sperm axoneme is incorporated into the zygote where its base associates with an aster near the male pronucleus that is known as the sperm aster. Later during mitosis, the incorporated sperm axoneme associates with one spindle pole^34,36^. This association with the aster and the spindle pole could be an indication that the axoneme-attached DC is functional.

**Fig. 6:**
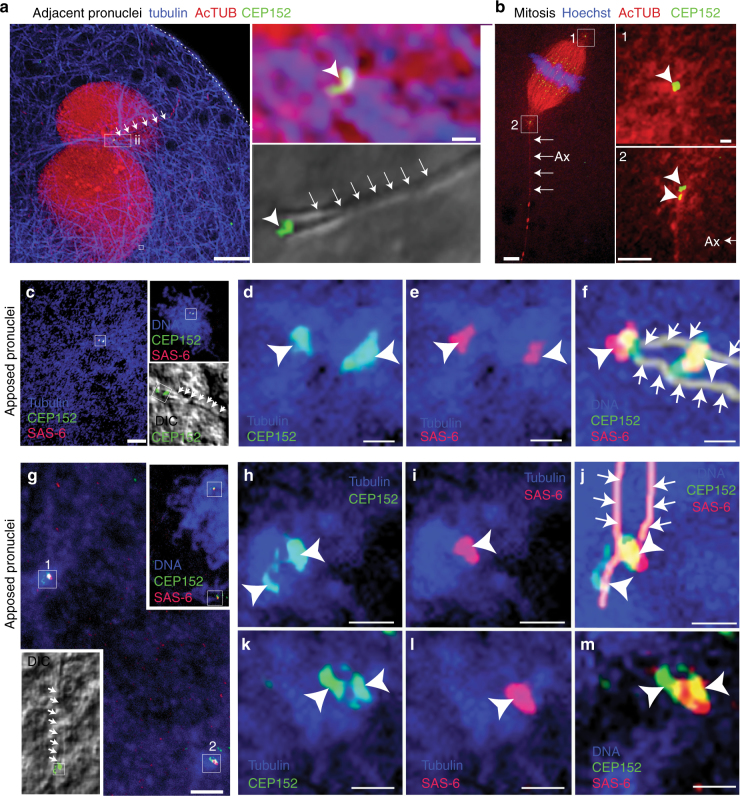
The remodeled DC is functional in bovine zygotes. **a**, **b** The axoneme with a CEP152-labeled DC was observed near the male pronucleus (**a**) and at the spindle poles (**b**). **a** Adjacent pronuclei (red), and the axoneme (arrows) at low magnification on the left. A pair of CEP152-labeled centrioles (arrowhead, right side high magnification) are found at the axoneme base. The bifurcated axoneme (arrows) is observable using DIC (lower right high magnification). **b** A spindle (red) with an attached axoneme (red, Ax, arrows) and a pair of CEP152-labeled centrioles (green, arrowheads, pole 2) at one pole and CEP152-labeled centrioles at the other pole (green, arrowhead, pole 1). Centrioles are within an aster (red). Scale bar 10 μm (left), and 1 μm (right). **c**–**f** Zygote with closely apposed pronuclei with centrioles retained at axoneme base has separated centrioles co-labeled with CEP152 (green) and SAS-6 (red) within a single aster (blue) (**f**). The upper inset in **c** shows the centrioles relative to the pronuclei (blue). The lower inset in **c** shows a DIC image of the axoneme (arrows) overlaid with CEP152-labeled centrioles (box, green). The CEP152 (green, arrowheads, **d**) and SAS-6-labeled centrioles are within an aster (blue, arrowheads, **e**). The composite image (**f**) shows the centrioles (arrowheads) at the bifurcated axoneme base (arrows). Scale bar 10 μm (left), and 1 μm (right). **g**–**m** A zygote with two separated centriole pairs (box “1” and “2” in **g** that each reside in an aster; blue). Upper inset in **g**, shows DNA (blue), and the labeled centrioles (boxes) relative to the pronucleus (blue). Lower inset in **g** shows a DIC image of the bifurcated axoneme (arrows) and CEP152-labeled centriole pair 1 (box). **h**–**j** Enlarged images of centriole pair 1 showing details of CEP152 (green, arrowheads, **h**) and SAS-6 (red, arrowheads, **j**) within the aster (blue). Image **j** shows centriole pair 1 (arrowheads) at the bifurcated axoneme base (arrows). **k**–**m** Enlarged images of centriole pair 2 showing CEP152 (green, arrowhead, **k**) and SAS-6 (red, arrowheads, **l**) within the aster (blue). Image (**m**) shows centriole pair 2 (arrowheads). Scale bar 10 μm (left), and 1 μm (right)

To test this, we examined the ability of the DC in bovine zygotes to recruit the PCM protein CEP152. In zygotes with adjacent male and female pronuclei, we found a pair of CEP152-labeled centrioles at the axoneme base, abutting a pronucleus. This suggests that the DC recruits PCM and forms a functional centrosome (Fig. 6a and Supplementary Fig. 7a–d). Later, in mitotic metaphase, we observed CEP152-labeled centrioles at both poles of the bovine spindle. At the pole with the attached sperm axoneme, we found a pair of CEP152-labeled centrioles (Fig. 6b and Supplementary Fig. 7c). This suggests that the DC also participates in spindle pole formation. Together, these findings suggest that the DC functions as a centriole in the zygote.

Similar to prior work, we observed by differential interference contrast (DIC) optics that the axoneme base nearest the paternal pronucleus often splays into two fibers^34,37^ (Fig. 6a). This axoneme base associates with the center of a microtubule aster, which splits later to generate a second aster (Supplementary Fig. 7d).

To determine if the DC was able to function as a platform for the formation of a new daughter centriole, we labeled zygotes with the daughter centriole protein SAS-6. We found that in zygotes with closely apposed male and female pronuclei the centrioles were retained at the bifurcated axoneme base (Fig. 6c and Supplementary Fig. 7d). These centrioles were co-labeled with CEP152 and SAS-6 within a single microtubule aster (Fig. 6c–f and Supplementary Fig. 7d). Later, when the sperm aster splits into two, we found a pair of centrioles at the center of each microtubule aster. Each of these pairs contains two CEP152 foci, only one of which is associated with a SAS-6 focus (Fig. 6g–m). One of the pairs is associated with the base of the bifurcated sperm axoneme (Fig. 6g–j and Supplementary Fig. 7d). Together, this suggests that centriole duplication occurs before aster duplication, and when it takes place a single daughter centriole forms in association with each of the sperm centrioles. Importantly, this means that the atypical DC is able to act as platform for the formation of a daughter centriole.

## Discussion

In summary, we have found that the mammalian sperm centrosome is remodeled during spermiogenesis. During the remodeling, centrosomal proteins were eliminated, reduced, or enriched. Many of these proteins were redistributed between the DC, PC, and PCM. The centriole distal lumen proteins were redistributed in the DC to form rods that associated with the splayed DC microtubules. As a result, the remodeled DC had a general appearance of truncated ovoid cone. The sperm provided the zygote with two centrioles, a typical centriole (the PC) and an atypical centriole attached to the axoneme base (the remodeled DC), as well as a remodeled, structural PCM, the striated columns and the capitulum. The DC was atypical but functional; in vitro, it recruited γ-tubulin. In vivo, the DC recruited PCM, formed a daughter centriole, and localized to one spindle pole in the zygote. Altogether, our findings support a paternal inheritance model where the sperm provides two functional centrioles, of varied structure and composition, along with the associated PCM/striated columns structures.

While our findings elucidate the origin of the zygote’s centrioles, they reveal a new enigma. The presence of a functional, yet atypical DC raises the question of the purpose of the remodeled structure. We have found previously that the *Drosophila* atypical centriole is essential for normal fertility and embryo development^15^, suggesting that the human atypical DC may be instrumental in fertility. The difference between the typical and atypical centrioles may provide a cue for early embryonic development^38^. Furthermore, it is possible that the remodeling step allows for some flexibility in the neck region for sperm movement.

Additionally, our work raises questions regarding the fate of the DC. If the DC remains attached to the bifurcated axoneme, as we have observed, it does not appear capable of forming a cilium. This may not be a problem as primary cilia form only in some cell fates (the epiblast) at much later stages (post-implantation)^39^. This work also has implications beyond the scope of reproductive biology; because several other differentiated cell types are thought to lack centrioles, it is possible that they have atypical centrioles^40,41^.

Mammalian and insect sperm were thought to carry a single functional centriole to the zygote. However, recently a second centriole that is atypical was described in insect sperm^15,16,25,42^. Similarly, here we show that mammalian sperm also have one typical and one atypical centriole. Together, these observations argue for an evolutionarily conserved sperm centriole number with variable structure, microtubule organization, and protein composition. This conservation suggests that sperm centrioles and their remodeling could play a critical role in fertility and early embryo development. Understanding the precise mechanisms of centrosome transmission during reproduction may help solve currently idiopathic forms of male infertility, generate novel targets for male contraception^43^, and even support organelle donation strategies to treat centriole-mediated infertility^44^.

## Methods

### Sperm preparation

Ejaculated spermatozoa were obtained from Coba Select Sires (bovine) and Manhattan Cryobank and Fairfax Cryobank (human). In all animals tested, motile ejaculated spermatozoa were washed and selected using a PureCeption density gradient according to instructions (Origio, ART-2004). All human sperm samples were acquired after approval from the University of Toledo’s Institutional Review Board and this work was declared exempt.

### U2OS cells

U2OS cell were obtained from Dr. Deborah Chadee (University of Toledo). They were grown in Dulbecco’s Modified Eagle’s Medium (DMEM; Mediatech), supplemented with 10% fetal calf serum (FCS; Atlanta Biologicals), at 37 °C in a humidified atmosphere supplemented with 5% CO_2_.

### Bovine cells and testes

TE11 p39 Bovine embryonic lung fibroblasts were obtained from Dr. Toshihiko Ezashi and Dr. R. Michael Roberts of the University of Missouri-Columbia. Bovine embryonic lung fibroblasts from 99-day fetuses were generated by Dr. Neil Talbot in 2009. They were grown in DMEM (Mediatech), supplemented with 10% FCS (Atlanta Biologicals), at 37 °C in a humidified atmosphere supplemented with 5% CO_2_. Bovine testes were purchased from Scholl’s slaughterhouse in Blissfield Michigan. They were dissected and embedded fresh in OCT (EMS Diasum 62550-01). The OCT-embedded testes were frozen on dry ice and then sectioned. The 10 μm sections were stained in the immunofluorescence protocol described below. As this work uses discarded tissue, no approval was required from University of Toledo’s Institutional Animal Care and Use Committee.

### Plasmids

POC1B312–406-His (amino acids 312–406 of NP_758440.1) in pQE10 vector were obtained from Dr. Chad Pearson^45^. POC1B312–406-GST was generated using the QuickFusion kit to move a POC1B312-406 fragment from the pQE10 vector into a pGEX-3× plasmid between restriction enzymes Xma1 and EcoR1.

### Protein purification

POC1B312–406 was grown in *E*. *coli* at 25 °C in lysogeny broth with 100 μg/mL ampicillin and then stored at 4 °C for 3 h to overnight; we then added 1 mM IPTG and incubated at 20 °C for 3 h. The bacteria were then centrifuged for 20 min at 12,000 × *g*, and the supernatant was discarded. The pellet was resuspended in lysis buffer (50 mM TRIS, 105 mM NaCl, 5% glycerol, and complete protease inhibitor cocktail (Roche)) and sonicated for 1 min at 10 s intervals at 40% power. The sonicated cells were then centrifuged at 20,000 × *g* for 30 min. POC1B312–406-His was purified by nickel beads, and POC1B312–406-GST was purified by glutathione bead affinity column. Purified protein was separated on an SDS-Page gel, and corresponding bands were excised and used to immunize rabbit and rats.

### Antibodies

Antibodies were generated against purified POC1B312–406-His. Polyclonal antibodies 537 and 538 were generated in rabbits by Pacific Immunology. Monoclonal antibodies (5G5A7 and 5G5A10) were generated in rat by ProMab using the purified POC1B312–406-His. Antibodies 537 and 538 were affinity purified by Pacific Immunology using purified POC1B312–406-GST. Antibodies 537 and 538 were used at a 1:100 dilution for immunofluorescence. The rat monoclonals were used at 1:20 for immunofluorescence. The polyclonal antibodies and rat monoclonal did not work well for western. All other antibodies used are described in Supplementary Table 1.

### Immunofluorescence

For immunofluorescence, 7 μL of PureCeption cleaned spermatozoa were placed on a poly-lysine slide (Sigma-Aldrich, P5899) and a Sigmacote coverslip (Sigma, SL2) was placed on top. The whole slide was then snap frozen and stored in liquid nitrogen. Sperm slides were stored in liquid nitrogen; when withdrawn, the coverslip was removed using forceps, and the slide was placed in a pre-chilled Coplin jar of ice-cold methanol for 2 min. Next, the slide was placed in 1× phosphate buffered saline (PBS) for 1 min, then placed for 60 min in fresh 1× PBS with 3% Triton X-1000 at room temperature. PBST-B was prepared by adding 1% bovine serum albumin (BSA) to PBST, and slides were then placed in PBST-B for 30 min. Primary antibodies diluted in PBST-B were added to slides at the concentrations listed in Supplementary Table 1, after which the slides were covered in parafilm, placed in a humidity chamber, and incubated at room temperature for 1 h or overnight at 4 °C. The slides were washed three times in PBST for 5 min each. Next, the secondary antibody mixture was prepared by combining PBST, secondary antibodies (see Supplementary Table 1B), and Hoechst 33258. The secondary antibody mixture was added to slides, and they were covered in parafilm and incubated for >1 h at room temperature. Slides were then washed three times with PBST for 5 min each, followed by three times with 1× PBS for 5 min each. Finally, the slides were sealed and imaged using a Leica Sp8 confocal microscope and some images (Fig. 4) were processed using a Leica HyVolution 2 System.

Sperm images were taken at a magnification of 640× and zoom of 6×, with 512 × 512 pixel density. Using Photoshop, immunofluorescence sperm images were cropped to 200 pixels by 100 pixels, or 100 pixels by 100 pixels. Immunofluorescence U2OS images were cropped to 150 pixels by 150 pixels or 25 pixels by 25 pixels. The intensity was modified to allow easy visualization, and the panels were resized to 300 DPI for publication. All photon counting was done with a constant laser power that, in various experiments, ranged between 0.25 and 2%, using CETN1/2, tubulin, or POC1B as a reference for centriole location.

Bovine zygote images in Fig. 4d were taken using a Nikon A1 four laser line confocal microscope equipped with elements acquisition and analyses software and were deconvolved. Multiple pictures of the bovine zygotes were taken, resulting in some bleaching in areas that were taken at higher zoom.

### Electron microscopy with high-pressure freezing

For TEM analysis of human sperm centrioles, the ejaculated sperm were centrifuged in Eppendorf tubes (1.5 mL) at 1000 rpm for 1 min, and then the pellets were processed using the high-pressure freezer system (Leica EM HPM100) in 20% BSA. The frozen samples were dehydrated and stained en bloc using a freeze substitution preprocessor (Leica EM AFS2) in 96% acetone with 1.5% OsO_4_ (osmium crystals dissolved in acetone and 4% water). The freeze substitution started with −90 °C for 6 h, then warmed from −90 to −10 °C over 15 h (5.3° slope). Then the samples were warmed to −3 °C over 1 h (7° slope) while being washed with 96% acetone (at −7 °C). They were then warmed from −3 to 4 °C over 1 h (7° slope) while being washed twice with 100% acetone. Lastly, the dehydrated samples were infiltrated and embedded in EMbed 812 resin while warming to room temperature. Ultrathin sectioning (70 nm) was performed using an ultramicrotome (Leica EM UC6), and sections were post-stained with 6% uranyl acetate (in 1:1 70% ethanol and 100% methanol), and Reynolds’ lead citrate (3–4% in preboiled double-distilled H_2_O). The sections were imaged using TEM (JEOL 1400-plus), operating at 80 kV.

### Correlative light and electron microscopy

Ejaculated sperm was obtained from Fairfax CryoBank, and cleaned using the PureCeption kit (Origio, ART-2004). Sperm cells were then immobilized on Poly L-lysine-coated coverslips, fixed with 1.5% glutaraldehyde in PBS, permeabilized with 0.3% Triton-X-100, and immunolabeled with POC1B antibody (as described above). The coverslip was mounted in the imaging chamber, 200-nm-thick Z-sections through the sperm cells were recorded to mark the position of fluorescent signals within the cells using a Nikon Eclipse Ti inverted microscope equipped with a 13-µm pixel DU888 camera (Andor) using a 100× NA 1.42 Plan Apo objective. High-magnification and low-magnification (100× and 20×) DIC images were additionally recorded to ascertain the position of sperm cells. Samples were then prepared for electron microscopy analysis according to the standard protocol. Briefly, samples were pre-stained with 2% osmium tetroxide and 1% uranyl acetate, dehydrated, and embedded in EMbed 812 resin. 80-nm-thick serial sections were made, post-stained with uranyl acetate and lead citrate, and imaged using a transmission electron microscope (Hitachi) operating at 80 kV. Image analysis and the alignment of the serial sections were performed using Fiji and Photoshop.

### Super resolution microscopy

3D-SIM data were acquired using an Elyra PS.1 microscope from Carl Zeiss, equipped with a plan-apochromat 63×/1.4 oil-immersion objective lens with an additional 1.6× optovar. Images were collected with an Andor iXon 885 EMCCD camera, resulting in a raw data pixel size of 79 nm. Z-stacks were acquired with a spacing of 101 nm/pixel. The fluorophores were excited with a 200-mW 488-nm laser. Images were acquired with a laser power, at the objective focal plane, of 52.6 mW, attenuated to 5%. Exposure times were between 50–200 ms and EMCCD camera gain values between 5–20. Five phases at each of three rotation angles (−75°, −15°, +45°) of the grid excitation pattern were acquired. A 495–550 band-pass filter was used to collect fluorescence from Alexa 488 antibody-labeled samples. A 570–620 band-pass filter was used to collect fluorescence excited with a wavelength of 555 nm. 5–8% power intensity was used. The data were processed using the SIM module of the Zen software, version 8.1, with Weiner filter between 10–3 and 10–5. Super resolution sperm images were cropped to 50 × 50 pixels.

Bovine sperm was attached to 25 mm, 1.5, high precision cover glasses (Warner Instruments), as described above for immunofluorescence. Anti-POC5 antibody (Thermo Fisher PA5-24308) was used at 1:70. CF647-conjugated FAB2 antibodies (Biotium) were used at 1:800 dilution to label primary antibodies. The anti-POC5 primary antibody was labeled by a Goat anti-rabbit Alexa 647 secondary antibody (Biotum) at 1:800 dilution. Before STORM imaging, samples were layered with 100 nm tetra-spectral fluorescent spheres (Invitrogen), which served as fiducial markers. Coverslips were mounted to Attofluor Cell chambers (Thermo Fisher) in imaging buffer (10% dextrose in 100 mM Tris at pH 8.0, 25 mM β-mercaptoethylamine, 0.5 mg/mL glucose oxidase, and 67 μg/mL catalase). 3D STORM imaging was performed on a Nikon N-STORM4.0 system using Eclipse Ti inverted microscope, Apo TIRF 100× SA NA 1.49 Plan Apo oil objective, 405, 561, 488, and 647 nm excitation laser launch (Agilent) and a back-illuminated EMCCD camera (Andor, DU897). The 647 nm laser line was used to promote fluorophore blinking. 405 nm laser was used to reactivate fluorophores. 561 nm laser was used to record the signals of fiducial markers. ~ 20,000 time points were acquired at a 50 Hz frame rate each 16–20 ms. NIS Elements (Nikon) was used to analyze and present the data.

STED images of bovine and human sperm were stained as described above and were acquired on a Leica SP8 with STED head at the University of Michigan.

### *Xenopus* egg extract

Studies with *Xenopus laevis* were conducted in compliance with the U.S. Department of Health and Human Services’ *Guide for the Care and Use of Laboratory Animals* and were approved by the University of Michigan Institutional Animal Care and Use Committee. *Xenopus* egg extract was generated according to the methods of Murray and Kirschner^46^, with modifications from Simerly et al.^34^ and Hannak and Heald^47^. In brief, *Xenopus laevis* females (obtained from Nasco) were induced to ovulate by injecting human chorionic gonadotropin (MP Biomedicals) into the dorsal lymph sac, and frogs were allowed to drop their eggs overnight in Ca^2+^-free 1× Marc’s Modified Ringer’s solution (MMR: 100 mM NaCl, 2 mM KCl, 1 mM MgCl_2_, 0.1 mM ethylene glycol tetraacetic acid (EGTA), 5 mM HEPES, pH 7.8). Eggs were collected and dejellied using 2% cysteine in Ca^2+^-free 1× MMR, pH 7.9, followed by washes in 50 mL each of extraction buffer (XB: 100 mM KCl, 1 mM MgCl_2_, 10 mM Hepes, 50 mM Sucrose, pH 7.7), XB-cytostatic factor (CSF) (200 mL XB, 200 μL 1 M MgCl_2_, 1 mL 1M K-EGTA), and XB-CSF with protease inhibitors (XB-CSF with 10 μg/mL each of leupeptin, chymostatin, and pepstatin). Eggs were then transferred to a 4-mL Beckman Ultra-Clear centrifuge tube (Beckman 344062), using a disposable plastic pipette with the tip cut off, taking care to make sure the eggs were not exposed to air. The 4-mL tube was placed inside a 13.2-mL Beckman Ultra-Clear centrifuge tube (Beckman 344059) as an adapter. The 4-mL tube was filled to the top with excess XB, and XB was added to the outside of the 4-mL tube so that the level of the XB outside the smaller tube matched the level of the eggs; this was done to prevent collapse of the tube during centrifugation. The tubes were centrifuged for 2 min at 1600 rpm at 16 °C in a clinical centrifuge to pack the eggs. Excess liquid was removed using a transfer pipette fitted with a 200-μL pipette tip. The tubes were then centrifuged at 11,500 rpm for 15 min at 16 °C using an SW41 hanging bucket rotor in a WX80 Sorvall ultracentrifuge to crush the eggs. The side of the tubes was pierced using an 18-gauge needle, and the straw-colored cytoplasmic layer was drawn into a 1-mL syringe, taking care to avoid the vesicles close to the lipids at the top of the cytoplasmic layer as well as the black nuclear layer below the cytoplasmic layer. The needle was removed from the syringe, and the cytoplasmic extract was transferred to an Eppendorf tube. The volume of the extract was estimated, then supplemented with 1:50 energy mix (190 mM creatine phosphate, 25 mM ATP, 25 mM MgCl_2_, 2.5 mM EGTA, pH 7.7), 1:1000 leupeptin, chymostatin, and pepstatin (10 mg/mL stock), 1:3000 nocodazole (6 mg/mL stock), and 1:500 cytochalasin B (5 mg/mL stock). This supplemented extract was stored for up to 2 h on ice prior to use. A “control buffer” was made by supplementing XB-CSF with the energy mix, protease inhibitors, nocodazole, and cytochalasin B.

### PCM recruitment in vitro

PCM recruitment was preformed according to the methods of Murray and Kirschner^46^ with modifications from Simerly et al^34^. In brief, 50–100 μL PureCeption-washed sperm (see sperm preparation) was placed onto a cleaned uncoated 12-mm circle glass coverslip (Fisher 12-545-81) and allowed to bind for 10–20 min. The unbound sperm was removed, and the bound sperm was permeabilized by adding 50–100 μL of 0.1% lysophosphatidyl choline (Sigma L4129-25MG) diluted in Kenney’s Modified Tyrode’s medium (KMT: 100 mM KCl, 2 mM MgCl_2_, 10 mM Tris-HCl, pH 7.0, and 5 mM EGTA) for 10 min. 50–100 μL of *Xenopus* cell-free extract or control buffer was added to the coverslip for 45–60 min at 37 °C. Coverslips were washed in KMT before being fixed in 20 °C cold methanol (5 min) and immunostained as described above. For the methanol pretreatments, the coverslips were fixed for 5 min in ice-cold methanol or 3.7% formaldehyde at room temperature after permeabilization and before the extract was added. Then they were washed in KMT before resuming the PCM recruitment protocol.

### Zygotes

Bovine in vitro fertilization and staining were performed as described previously^36,48^. In brief, bovine oocytes were acquired from a slaughterhouse and fertilized by Applied Reproductive Technology (AppliedReproTech). As this work uses commercially available cells, approval was not required from University of Toledo’s Institutional Animal Care and Use Committee. They were shipped in fertilization media (supplied by AppliedReproTech) overnight in an incubator at 39 °C. Upon receipt, cumulus cells were removed using a stripper pipette with 135-μm tips (Origio MXL3-135) in the D1 growth media (provided by AppliedReproTech). Then cumulus-free zygotes were cultured at 39 °C with 5% CO_2_ until a few zygotes divided (around 28–30 h). Zonae pellucidae were removed by soaking them in a few mL 2% Pronase (Sigma 10165921001) in human tubal fluid (HTF) for less than 2 min and were then fixed and prepared according to the methods of Simerly and Schatten^48^. In brief, zona-free zygotes were dropped onto a poly-lysine 22-mm square coverslip immersed in protein-free, calcium-free HTF (the coverslip sits in a 6-well dish, with the well full of HTF). We removed most of the HTF using a pipette and replaced it with either 2% paraformaldehyde in HTF or Buffer M (enough to fill the well), followed by ice-cold methanol (enough to fill the well) for 10–20 min.

### Statistical methods

Experiments were repeated at least three times (*N* > 3), and statistical analyses (average +/− standard deviation) were done with Excel. A two-tailed, unpaired Student’s *t*-test was used to determine *P*-value (*P*). Chi-squared tests were done as indicated where categorical data was available. ANOVA was done in SPSS with Fisher’s LSD post hoc. *P*-value designations are: *P < 0.05, **P < 0.01, and ***P < 0.001.

### Data availability

All data generated or analyzed during this study are included in this published article (and its Supplementary Information files) or are available from the authors.

## Electronic supplementary material

Supplementary Information
