# Author Correction: A novel atypical sperm centriole is functional during human fertilization

**DOI:** 10.1038/s41467-018-05324-z

**Published:** 2018-07-13

**Authors:** Emily L. Fishman, Kyoung Jo, Quynh P. H. Nguyen, Dong Kong, Rachel Royfman, Anthony R. Cekic, Sushil Khanal, Ann L. Miller, Calvin Simerly, Gerald Schatten, Jadranka Loncarek, Vito Mennella, Tomer Avidor-Reiss

**Affiliations:** 1 Department of Biological Sciences, University of Toledo, 2801W. Bancroft, Toledo, OH 43607 USA; 2 Cell Biology Program, The Hospital for Sick Children, Department of Biochemistry, University of Toronto, 555 University Avenue, Toronto, ON M5G 1X8 Canada; 3 Laboratory of Protein Dynamics and Signaling, Center for Cancer Research, National Cancer Institute, 1050 Boyles Street, Frederick, MD 21702 USA; 4 Department of Molecular, Cellular, and Developmental Biology, University of Michigan, 830 North University Ave, Ann Arbor, MI 48109 USA; 5 Departments of Cell Biology; Obstetrics, Gynecology and Reproductive Sciences; and Bioengineering, Magee-Womens Research Institute, University of Pittsburgh School of Medicine, 204 Craft Avenue, Pittsburgh, PA 15213 USA

Correction to: *Nature Communications* ; 10.1038/s41467-018-04678-8; published online 09 June 2018

In the original version of this Article, the affiliation details for Jadranka Loncarek and Vito Mennella were incorrectly given as ‘Cell Biology Program, The Hospital for Sick Children, Department of Biochemistry, University of Toronto, 555 University Avenue, Toronto, ON, M5G 1X8, Canada’ and ‘Laboratory of Protein Dynamics and Signaling, Center for Cancer Research, National Cancer Institute, 1050 Boyles Street, Frederick, MD, 21702, USA’, respectively. This has now been corrected in both the PDF and HTML versions of the Article.

