# Supplementary material for: A novel atypical sperm centriole is functional during human fertilization
Source: Nat Commun. 2018 Jun 7;9:2210. doi: 10.1038/s41467-018-04678-8 (PMC5992222; doi:10.1038/s41467-018-04678-8)
Supplement: Supplementary file 1 — Supplementary Information [file 41467_2018_4678_MOESM1_ESM.pdf]

A Novel Atypical Sperm Centriole is Functional During Human Fertilization

Fishman et al.

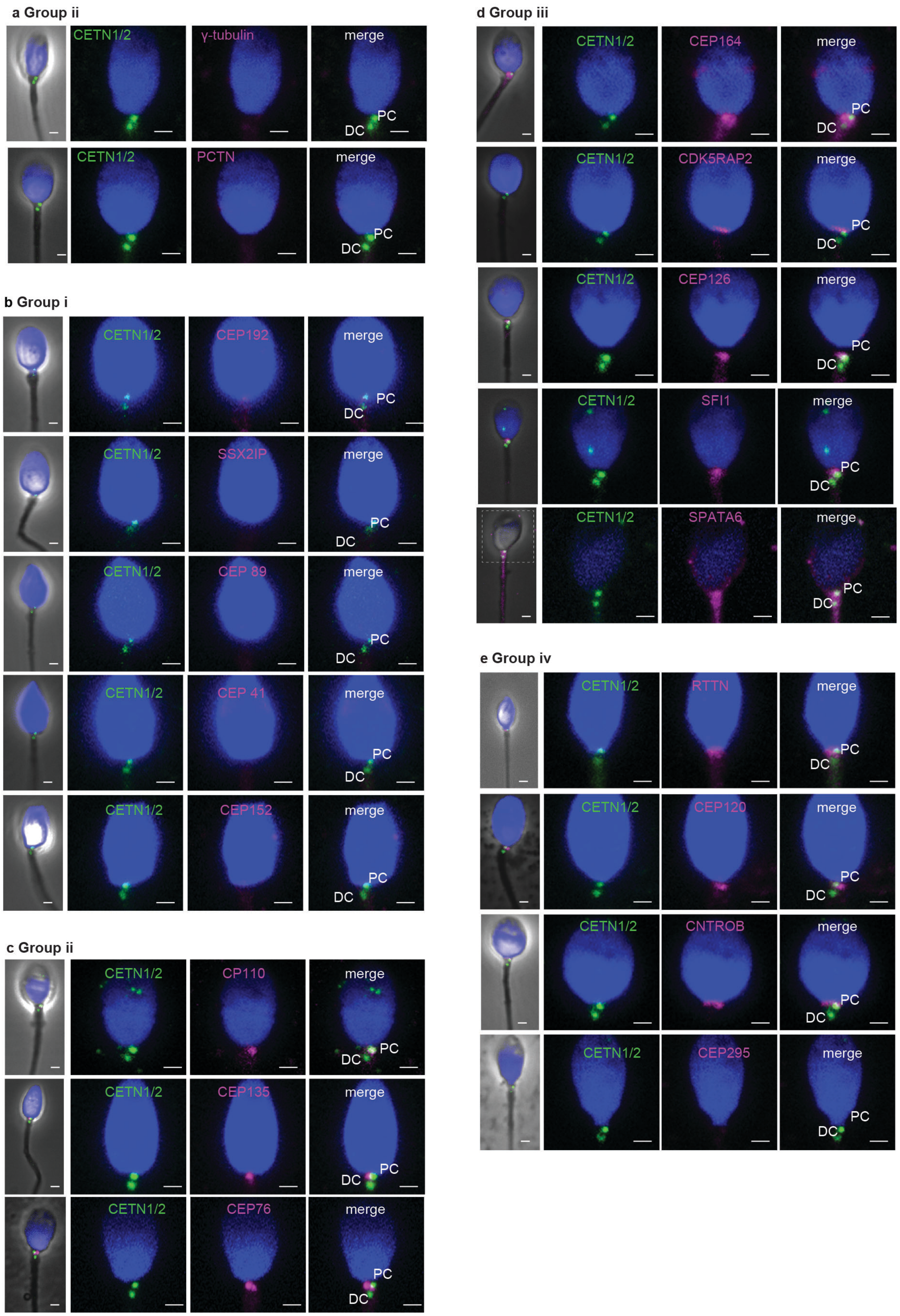
a Group ii
CETN1/2
γ-tubulin
merge
PC
DC
CETN1/2
PCTN
merge
PC
DC
b Group i
CETN1/2
CEP192
merge
PC
DC
CETN1/2
SSX2IP
merge
PC
DC
CETN1/2
CEP 89
merge
PC
DC
CETN1/2
CEP 41
merge
PC
DC
CETN1/2
CEP152
merge
PC
DC
c Group ii
CETN1/2
CP110
merge
PC
DC
CETN1/2
CEP135
merge
PC
DC
CETN1/2
CEP76
merge
PC
DC
d Group iii
CETN1/2
CEP164
merge
PC
DC
CETN1/2
CDK5RAP2
merge
PC
DC
CETN1/2
CEP126
merge
PC
DC
CETN1/2
SFI1
merge
PC
DC
CETN1/2
SPATA6
merge
PC
DC
e Group iv
CETN1/2
RTTN
merge
PC
DC
CETN1/2
CEP120
merge
PC
DC
CETN1/2
CNTROB
merge
PC
DC
CETN1/2
CEP295
merge
PC
DC

Supplementary Figure 1: Proteins that localize to the sperm neck but not the DC

a) As previously described[1] the PCM proteins PCNT and γ-tubulin were undetectable (group ii), but the centriolar protein CETN1/2 reliably labeled the DC and PC in ejaculated spermatozoa (group i). CETN1/2 DC to PC ratio is 1.14 ± 0.2, n=6.

b) Antibodies against CP110, CEP135, and CEP76 labeled near the PC (group i).

c) Antibodies against CEP192, SSX2IP, CEP89, CEP295, CEP41, and CEP152 did not specifically label the sperm neck (group ii). The antibodies for these proteins either did not label the sperm neck at all or labeled the neck and the midpiece equally. Gamma tubulin and PCNT (**Supplementary Fig 1a**) also are part of this group.

d) Antibodies against SPATA6, CEP164, CDK5RAP2, RTTN, CEP120, SFI1, CEP126, and CNTROB labeled the sperm neck where the striated columns and/or capitulum are found (group iii). The antibodies for these proteins labeled a line, ring, or partial ring at the base of the nucleus, external to the CETN1/2-labeled PC tip (i.e., the antibody labels adjacent to the PC, but the focus is on the outside of the sperm when compared to the axis of the DC/axoneme), or broadly labeled most of the sperm neck.

e) Antibodies against centriolar proteins RTTN and CEP120 mark the capitulum and the centriolar protein CEP295 is absent altogether (group iv). This group contains centriolar proteins that unexpectedly localize to the capitulum or are unexpectedly absent from the PC.

Scale bars 1 μm

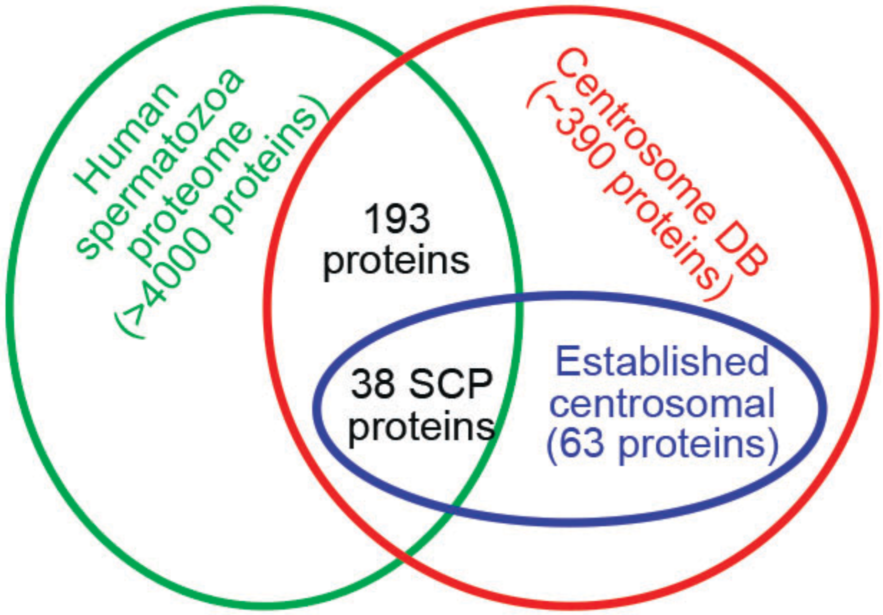

Supplementary Figure 2: Some Centrosomal Proteins are Found in Human Spermatozoa Proteomes

Comparison of human spermatozoa proteomes (as determined by MS; Mass Spectrometry)[2-5] (green) to the centrosome:db (database) (red) and to a list of established centrosomal proteins (blue) resulted in 193 candidate spermatozoan centrosomal proteins and 38 candidate established centrosomal proteins that are listed below in **Supplementary Table 1**.

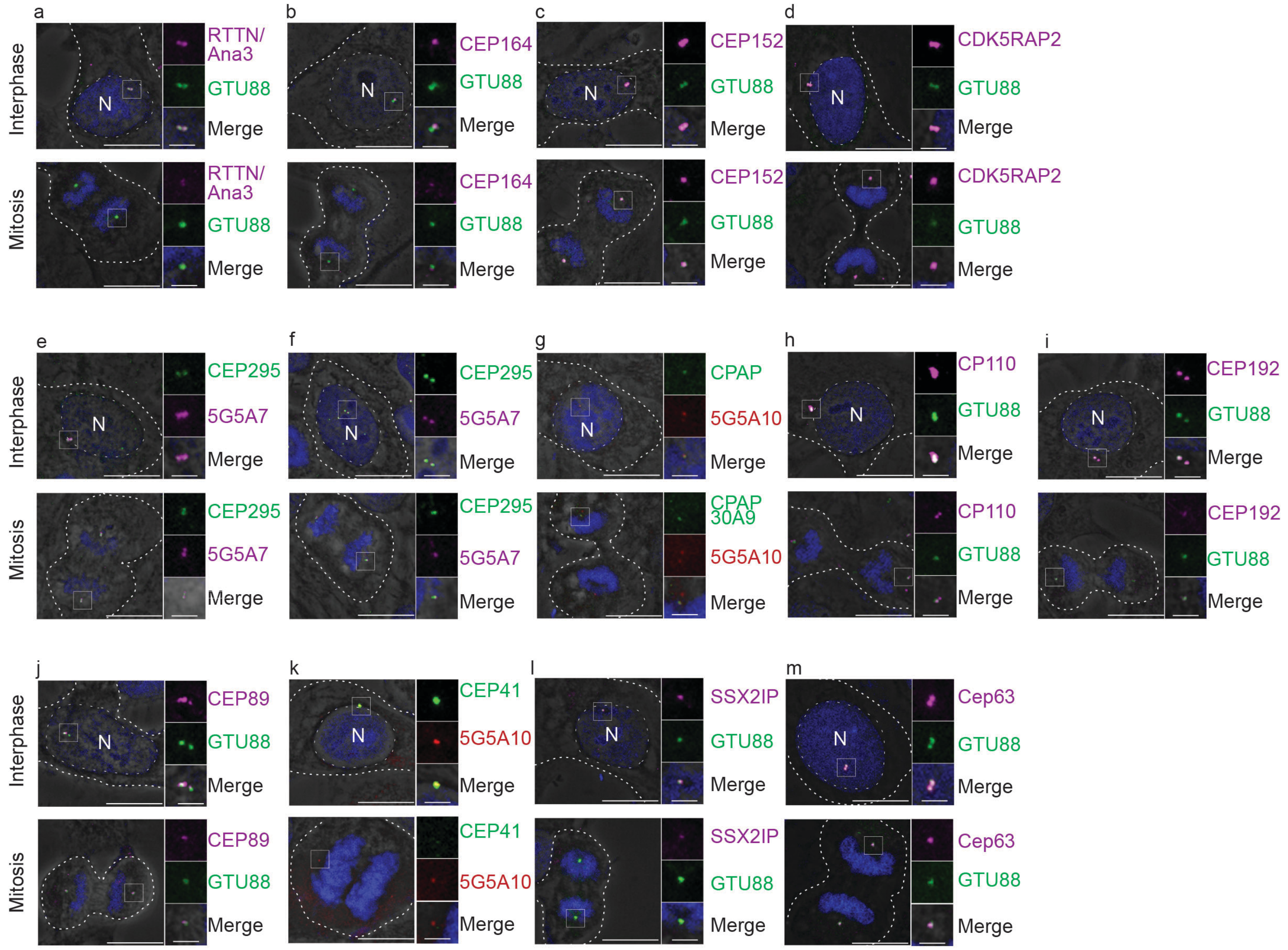

Supplementary Figure 3: Centrosomal antibodies used stained the centrosome of U2OS cells

a-e) The antibodies used in this study were tested and verified by staining the centrosome of U2OS cells. Centrosomal staining was observed in interphase and/or mitosis and was determined by colocalization with a common marker for the centrosome, anti γ-tubulin antibody (GTU88) or POC1B antibodies (clone 5G5A7 or 5G5A10).

Note that CEP164(**b**), CEP192 (**i**), CEP89(**j**), and SSX2IP(**l**) labeled the centrioles strongly during interphase, but only very weakly labeled the centrioles during mitosis.

Scale bar 10μm in low magnification images (left), and 2μm in centriole high-magnification images (right).

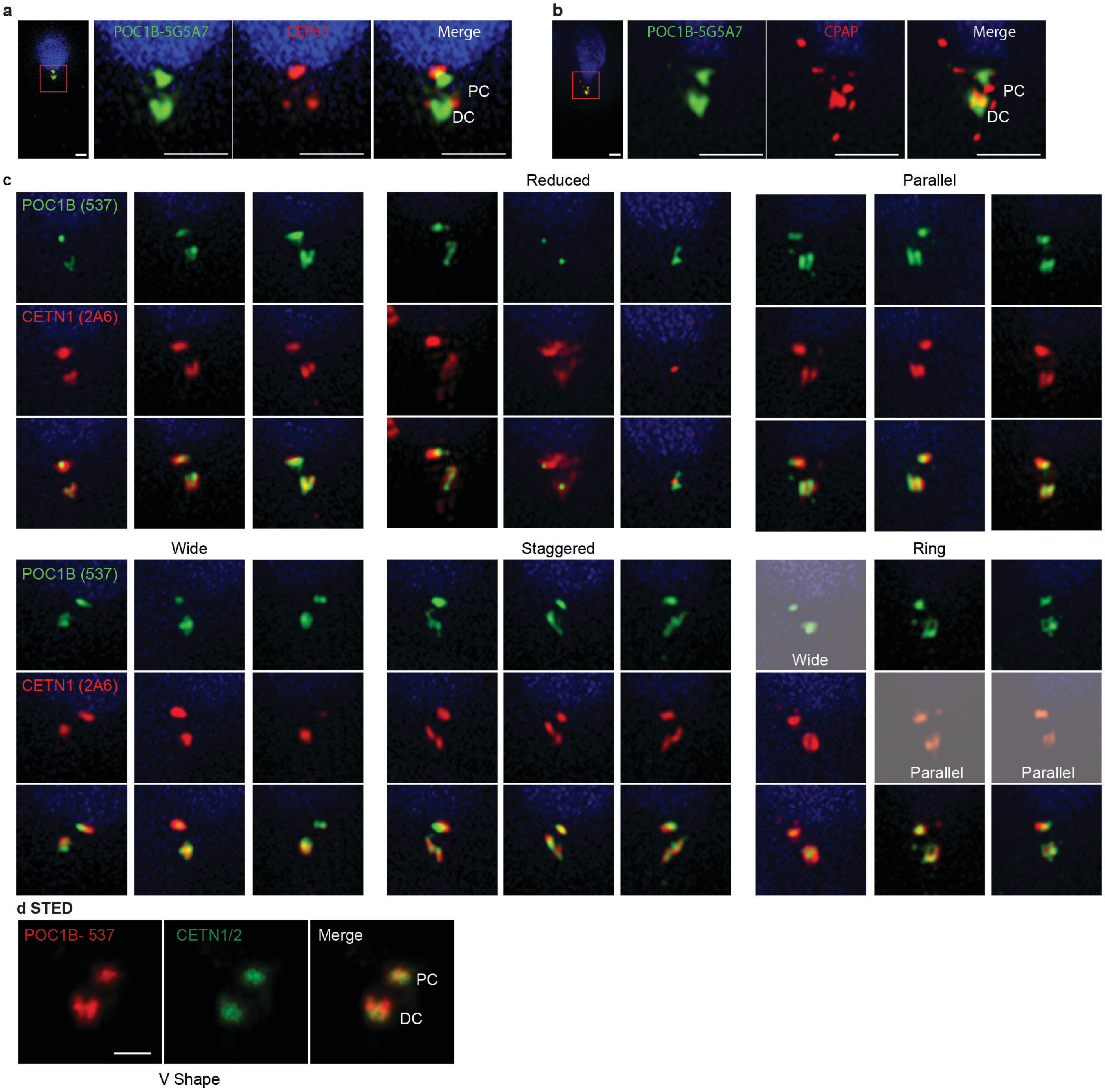

a
POC1B-5G5A7
CEP63
Merge
PC
DC
b
POC1B-5G5A7
CPAP
Merge
PC
DC
c
Reduced
Parallel
POC1B (537)
CETN1 (2A6)
Wide
Staggered
Ring
POC1B (537)
CETN1 (2A6)
Wide
Parallel
Parallel
d STED
POC1B- 537
CETN1/2
Merge
PC
DC
V Shape

Supplementary Figure 4: The most common morphology of the DC proteins is a V shape

a-b) 3D-SIM showed V orientation of centriolar protein POC1B; CEP63 appeared as a focus at the base of each rod; and CPAP was diffused around the two rods.

c) Representative figures of the six types of DC rod morphologies. Note that in most types, except for the ring type, CETN1 and POC1B have the same morphology, but in the ring type, CETN1 and POC1B morphologies did not always match; the pictures with the inconsistent type are greyed-out, and the inconsistent type is identified in white letters.

d) STED showed V orientation of centriolar proteins POC1B and CETN1/2.

Scale bars 1 μm

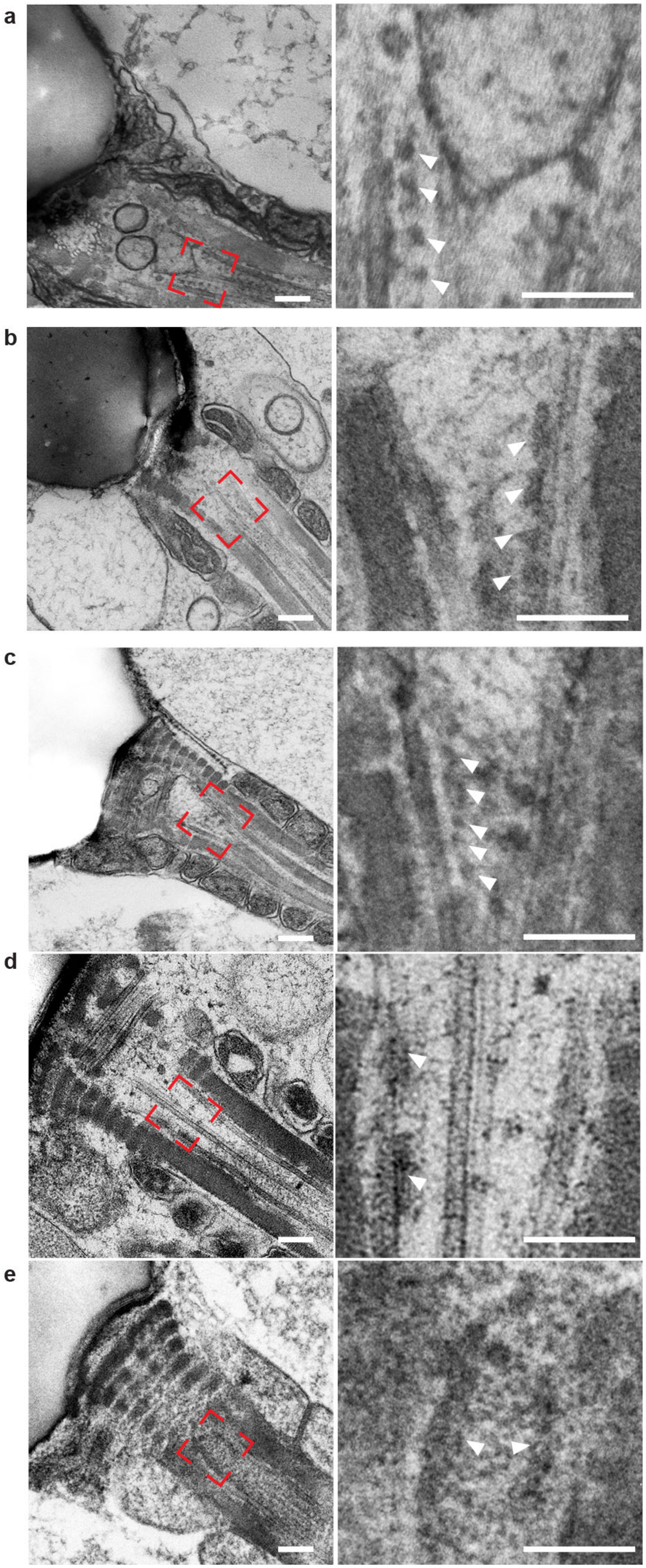
a
b
c
d
e

Supplementary Figure 5: A possible structural basis for the DC rods

a-e) Examples of longitudinal sections that showed the splayed DC microtubules near electron-dense material (white arrowheads) that might be the rods. Scale bar 200nm in low magnification images (left), and 100nm in centriole high-magnification images (right).

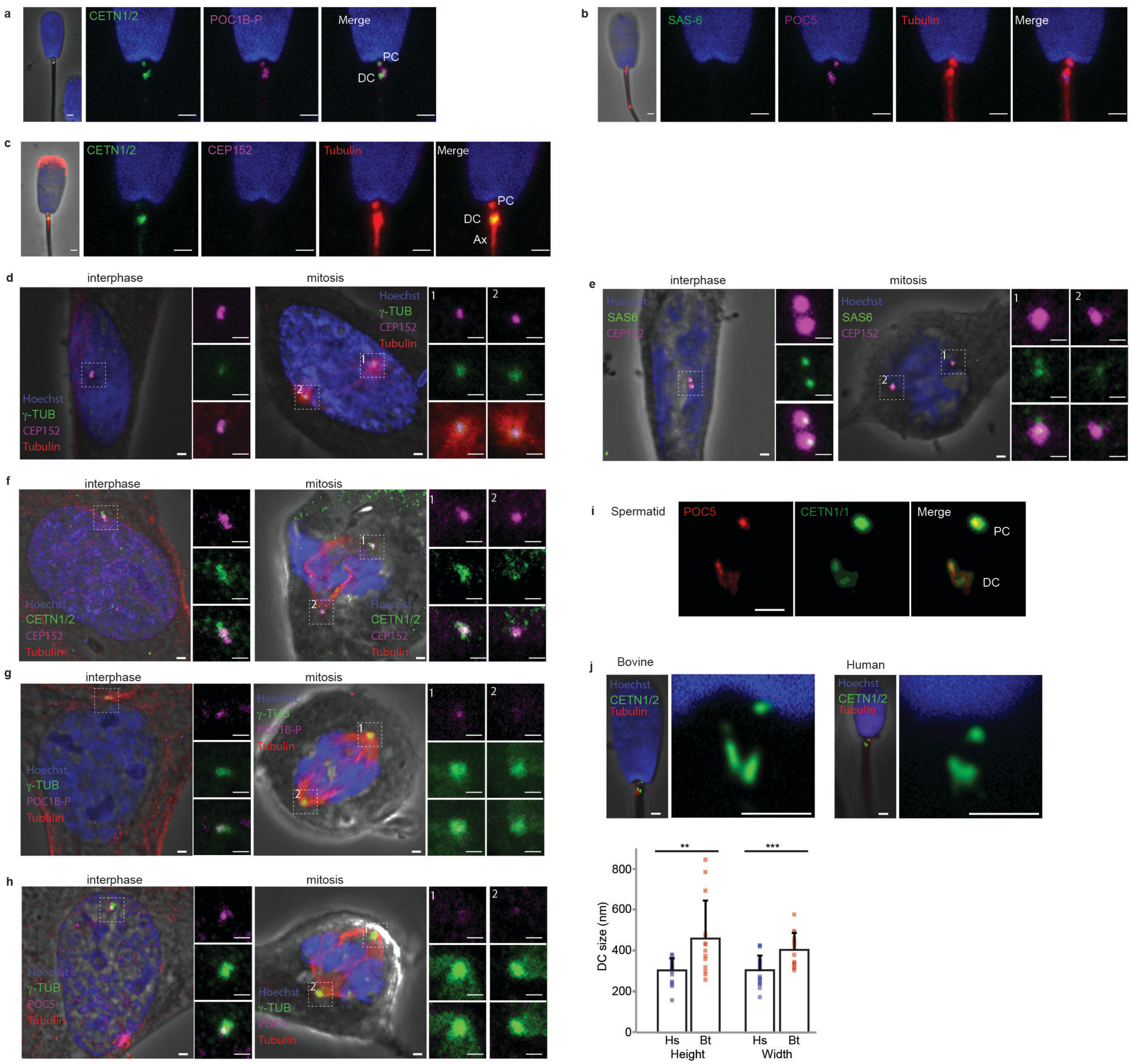
a
CETN1/2
POC1B-P
Merge
PC
DC
b
SAS-6
POC5
Tubulin
Merge
c
CETN1/2
CEP152
Tubulin
Merge
PC
DC
Ax
d
interphase
mitosis
Hoechst
γ-TUB
CEP152
Tubulin
e
interphase
mitosis
Hoechst
SAS6
CEP152
f
interphase
mitosis
Hoechst
CETN1/2
CEP152
Tubulin
g
interphase
mitosis
Hoechst
γ-TUB
POC1B-P
Tubulin
h
interphase
mitosis
Hoechst
γ-TUB
POC5
Tubulin
i
Spermatid
POC5
CETN1/1
Merge
PC
DC
j
Bovine
Human
Hoechst
CETN1/2
Tubulin
DC size (nm)
800
600
400
200
0
**
***
Hs
Bt
Height
Width

Supplementary Figure 6: Antibodies against Centriolar Proteins label to Bovine cells

a) CETN1/2 and POC1B-P antibodies recognized both the PC and the DC in bovine spermatozoa – both recognized the DC more strongly than the PC.

b) SAS-6 antibody did not label the bovine ejaculated spermatozoa, but POC5 antibody recognized both the PC and the DC. Sheep anti-tubulin antibody (Cytoskeleton) recognized both centrioles and the axoneme.

c) CEP152 antibody did not label the bovine ejaculated spermatozoa. Sheep anti-tubulin antibody recognized both centrioles and the axoneme.

d-h) During interphase (left) and mitosis (right) in TE11 p39 bovine cells, at the centrioles in the center of the aster (tubulin), CEP152 antibody colocalized with γ-tubulin (GTU88) (**d**), SAS-6 (**e**), and CETN1/2 (**f**), and γ-tubulin (GTU88) colocalized with POC1B (**g**) and POC5 (**h**).

i) STED showed rods of centriolar proteins POC5 and CETN1/2.

j) HyVolution Confocal Microscopy shows the size difference between DC CETN1/2 in human spermatozoa and bovine spermatozoa. The left panels are projections; the right panels are single z slice. Hs; humans, Bt; Bovine

Scale bars 1 μm. Error bars represent +1 standard deviation.

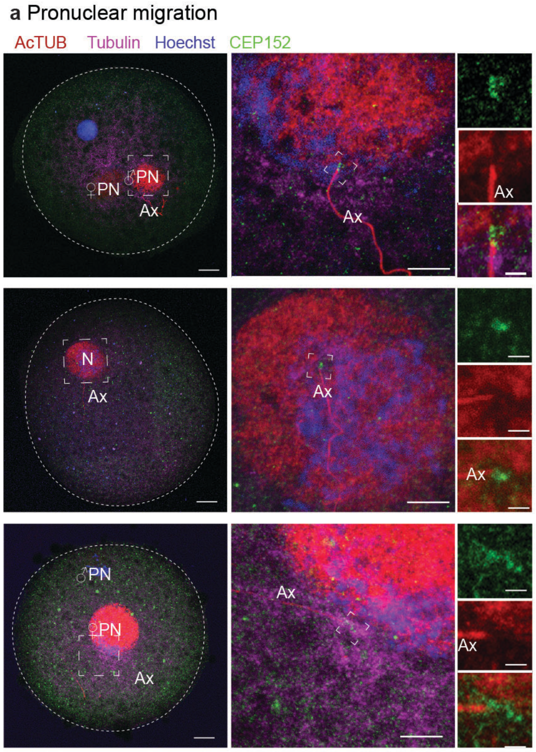

b CEP152 is recruited to the axoneme base

Stage	(i) Pronuclei configuration (n)	(ii) CEP152 foci number near the axoneme base no foci	1 focus	1 pair (2 foci)	multiple foci (>3)	2 pairs (or 4 foci)
Early	Apart (15)	3/15 (20%)	1/15 (7%)	10/15 (67%)	0/15 (0%)	1/15 (7%)
	Adjacent (23)	3/23 (13%)	3/23 (13%)	12/23 (52%)	4/23 (17%)	1/23 (4%)
	Apposed (65)	4/65 (6%)	12/65 (18%)	32/65 (49%)	10/65 (15%)	7/65 (11%)
Late	Mitotic (18)	1/18 (6%)	0/18 (0%)	9/18 (50%)	3/18 (17%)	5/18 (28%)

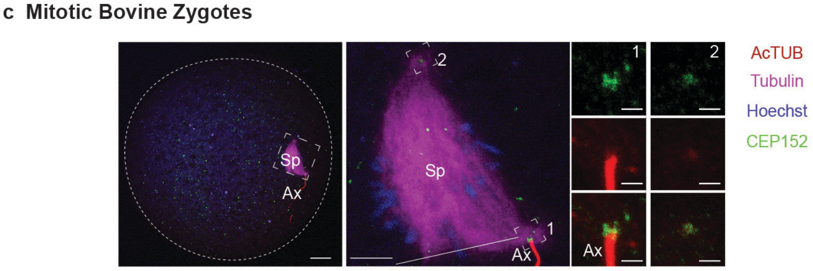

d The DC is able to act as a platform for the formation of a new daughter centriole

Stage	(i) Pronuclei configuration (n)	(ii) Aster number 1	(ii) Aster number 2	(iii) CEP152 foci position Together with sperm tail	(iii) Separated	(iv) CEP152 foci number 1 focus	(iv) 2 foci	(iv) 3 or 4 foci	(v) SAS6 foci associated with CEP152 No foci	(v) 1 focus	(v) 2 foci
Early	Apart (4)	4/4 (100%)	0/4 (0%)	4/4 (100%)	0/4 (0%)	2/4 (50%)	2/4 (50%)	0/4 (0%)	4/4 (100%)	0/4 (0%)	0/4 (0%)
	Adjacent (6)	3/6 (50%)	3/6 (50%)	5/6 (83%)	1/6 (17%)	1/6 (17%)	1/6 (17%)	4/6 (67%)	5/6 (83%)	1/6 (17%)	0/6 (0%)
Late	Apposed (38)	16/38 (42%)	22/38 (58%)	17/38 (45%)	21/38 (55%)	0/38 (0%)	7/38 (18%)	31/38 (82%)	16/38 (42%)	12/38 (32%)	10/38 (26%)
conclusions:	Pronuclei migrate towards each other	Asters split/duplicate		A centriole separates and moves away from sperm tail		Centrioles duplicate			A new centriole forms near the DC		

Supplementary Figure 7: The remodeled DC recruits CEP152 and forms a daughter centriole

a) The axoneme base was labeled by CEP152 during pronuclear migration in bovine zygotes. Scale bars on far left, low magnification images are 10 μm, middle images are 5 μm, and far right, high magnification images are 1 μm.

b) Analysis of 121 bovine zygotes between 28-32 hours post fertilization demonstrates that at any stage (i) most (>80%) zygotes have at least two CEP152 foci associated with the base of the sperm axoneme (ii).

c) The axoneme base was labeled by CEP152 and was connected to a spindle pole during mitosis in bovine zygotes. Scale bars on far left, low magnification images are 10 μm, middle images are 5 μm, and far right, high magnification images are 1 μm.

d) Analysis of 48 bovine zygotes between 28-32 hours post fertilization at three pronuclei configurations (Apart, Adjacent, and Apposed, i). We found that change in the number of microtubule asters (ii), from 1 to 2, begins when the male and female pronuclei are adjacent. At the same time, the CEP152 labeled centrioles separated from each other (iii). As the pronuclei appose, the number of CEP152 foci in the cell increases, indicating that the centrioles have duplicated (iv). Furthermore, SAS-6, a protein necessary for centriole duplication, is overwhelming detected near CEP152 only after male and female pronuclei appose in the cytoplasm.

Pink text highlights the changes observed in transition from early to late stages. ♀PN, female pronucleus; ♂PN, male pronucleus; Ax, axoneme.

	Protein	Uniprot ID	Mass-spec reference (% Coverage if available)	U2OS staining	Sperm staining	Ab Dilution	Ab source
Centriolar	CEP135	Q66GS9	[5](23%); [4](5%); [2]	Not tested	DC<<PC	1:800	Dr. Tang K Tang[6]
	POC1B	Q8TC44	[5](64%/31%); [4](13%); [2]	Yes<br>No<br>No<br>Yes<br>Yes	DC>PC<br>No<br>DC>PC<br>DC>PC<br>DC>PC	1:400<br>1:100<br>1:100<br>1:20<br>1:20	Dr. Chad Pearson[7]<br>Dr. Andrew Fry[8]<br>Avidor-Reiss 537<br>Avidor-Reiss Clone 5G5A7<br>Avidor-Reiss Clone 5G5A10
	CETN1	Q12798	[5](39%); [2]	Yes<br><br>Not tested	DC<=PC<br><br>DC<=PC	Lot dependent<br>1:10	Millipore C# 04-1624 Clone 20H5<br><br>Santa cruz C#sc-293494 Clone 2A6
	CEP76	Q8TAP6	[5](23%)	Not tested	DC<=PC	1:100	Dr. Brian Dynlacht[9]
	CEP295/ KIAA1731	Q9C0D2	[2]	Yes<br>Yes	No<br>No	1:100<br>1:100	Sigma C# HPA038596<br>Dr. Tang K Tang[10]
	CPAP/CENPJ	Q9HC77	[2]	Yes	PCM*	1:5	Dr. Jay Gopalakrishnan (clone 30A9)[11]
	CP110/CNTRL	O43303	[5](5%); [3]	No<br>Yes	No<br>DC<<PC	1:100<br>1:100	Dr. Stephen Doxsey[12]<br>Proteintech C#12780-1-AP
	POC1A	Q8NBT0	[5](17%)	No	No	1:100	Dr. Andrew Fry[8]
	NA14/DIP13	O43805	[5](25%); [3]	No	No	1:10	Santa Cruz C#sc-376254
	CEP120	Q7TSG1	[5](4%)	Not tested	capitulum	1:200	Dr. Tang K Tang[6]
	TSGA10	Q9BZW7	[5](47%);[2]				
	ANA3/RTTN	Q86VV8	[5](3%)	Yes	capitulum	1:100	Dr Jordan Raff[13]
	CEP70/BITE	Q8NHQ1	[5] (28%); [4](4%)				
	PLK4	O00444	[3]				
	CENTRIN 2	P41208	[5](27%)	Yes	DC<=PC	Lot dependent	Millipore C# 04-1624 Clone 20H5
	POC5	Q8NA72	[5](7%)	Yes<br>Not Tested<br>Yes	DC=PC<br>DC=PC*<br>DC=PC	1:500<br>1:100<br>1:200	Dr. Michel Bornens[14]<br>Bethyl laboratories C# A303-340A<br>Thermo Fisher PA5-24308
	SPATC1/ Speriolin	Q76KD6	[5](34%); [4](8%); [2]				
	LGALS3BP	Q08380	[5](17%)				
	CEP44/ KIAA1712	Q9C0F1	[5](3%)	No	No	1:100	Bethyl Laboratories C# A304-955A
	CEP78	Q5JTW2	[5](1%)	No	No	1:100	Dr. William Tsang[15]
	POC18/WDR67	Q96DN5	[5](7%)				
	ATF-5/ATFX	Q9Y2D1	[3]				
	CEP126	Q9P2H0	[3]	Not tested	capitulum	1:100	Sigma C#HPA038399
	sfi1	A8K8P3	[3]	No	capitulum	1:100	Santa Cruz C#sc-86859
	OFD1			Not Tested	PC and below DC<br>(not shown)	1:100	Dr. Jeremy Reiter [16]
	CEP131	Q9UPN4	NA	Not Tested	No	1:100	Dr. Jeremy Reiter [17]
	CENTRIN 3	O15182	NA	No	No	1:10	Santa cruz C#sc-365697
	STIL	Q15468	NA	No	No	1:10	Santa cruz C#sc-271910
	SPICE1	Q8N0Z3	NA	Not tested<br><br>Not tested	Diffuse centriolar region staining (not shown)<br>Diffuse centriolar region staining (not shown)	1:500<br><br>1:100	Dr. Jens Luders[18]<br><br>Thermo Fisher C#PA5-64185
	SAS6	Q6UVJ0	NA	No **but does work in bovine cells<br>Not tested	No<br><br>No	1:10<br><br>1:100	Santa cruz C#sc-81431<br><br>Dr. Pierre Gocnzy[19]
	CEP104	O60308	NA	No	No	1:10	Santa cruz C#sc-514475
	CEP128/ LEDP/13	Q6ZU80	NA				
	CEP19	Q96LK0	NA				
	CEP97	Q8IW35	NA				
	FGFR1OP/FOP	O95684	NA				
	CNTROB	Q8N137	NA	No	capitulum	1:100	Dr. František Liška [20]
	NINEIN	Q8N4C6	NA				

**PCM**	CEP152	O94986	[2,3]	Not tested<br>Yes<br>Not tested<br>Not tested<br>Not tested	No<br>No<br>No<br>No<br>No	1:100<br>1:100<br>1:100<br>1:100<br>1:100	Dr. Ingrid Hoffman [21]<br>Dr. Kyung Lee [22]<br>Abverify<br>Dr. Erich Nigg[23]<br>Bethyl Laboratories, C# A302-480A
	CEP63	Q96MT8	[5](2%)	Not tested<br>Yes	DC<PC<br>DC<PC	1:100<br>1:800	Dr. Fanni Gergley [24]<br>Millipore C# 06-1292
	CEP215/CDK5rap2	Q96SN8	[3]	Yes	capitulum	1:100	Dr. Robert Qi[25]
	pericentrin	O95613	[2]	Yes	No	1:100	Bethyl Laboratories C# A301-348A
	CEP192/SPD2	Q8TEP8	NA	Yes	No	1:100	Dr. David Sharp [26]
	CEP57	Q86XR8	NA				
	PLK1//2//3	P53350<br>Q9NYY3<br>Q9H4B4	NA	No	No	1:15	Santa Cruz C# sc-17783
**Other location in the Centrosome**	CEP164	Q9UPV0	[5](2%)	Yes	Striated Columns	1:100	Dr. Gislene Pereira[27]
	ODF2/Cenexin	Q5BJF6	[5](64%/53%); [4](40%); [2]; [3]				
	CLERC/VFL1	Q9C099	[4]; [2]; [5](40%)				
	TSGA14/CEP41	Q9BYV8	[5](5%)	Yes	No	1:100	Dr. Joseph Gleeson[28]
	BUG14/WDR16	Q8N1V2	[5](51%); [4](32%); [2]				
	CEP83/CCDC41	Q9Y592	[2]				
	DCTN1/P135	Q14203	[3]; [5] (48%)				
	ODF1	Q14990	[4]; [5](47%)				
	SSX2IP	Q9Y2D8	[5](6%)	Yes	capitulum	1:100	Dr. Oliver Gruss [29]
	PCM1	Q15154	NA	Yes	No	1:400	Santa Cruz C#sc-398365
	CEP170/FAM68A	Q5SW79	NA				
	CEP89/CCDC123	Q96ST8	NA	Yes	No	1:100	Dr. Michel Bornens[30]
	NINL/NLP	Q9Y2I6	NA				
	SCLT1/CAP1A	Q96NL6	NA				
	CEP290	O15078	NA	Not tested	ring in DC	1:200	Abcam C#84870
	KIAA0753/OFIP	Q2KHM9	NA				
	FBF1	Q8TES7	NA	Not tested	No	1:100	Thermo Fisher C# PA5-54864
**Sperm proteins**	SPAG4	Q9NPE6	NA	No	No	1:50	Dr. Frans Van Der Hoorn[31]
	SPATA6	Q9NWH7	NA	No	Striated columns	1:600	Dr. Ryuzo Yanagimachi[32]
**Tubulin**	Alpha tubulin	NA	NA	Yes (not shown)	DC=PC*	1:100	Sigma clone DM1A
	Beta tubulin	NA	NA	Yes (not shown)	DC=PC*	1:100	Hybridoma Bank clone E7
	Acetylated tubulin	NA	NA	Yes (not shown)	DC=PC*	1:200	Sigma clone 6-11B-1
	Gamma tubulin	NA	NA	Yes (not shown)	No	1:100	Sigma clone GTU88
	Tubulin (sheep)	NA	NA	Yes (not shown)	DC=PC*	1:1200	Cytoskeleton C#ATN02
	Tubulin (rat)	NA	NA	Not tested	Not tested	1:400 in zygotes	Millipore clone YOL134

Supplementary Table 1: Tested Centrosomal Proteins and Antibodies

The table shows the 63 established centrosomal proteins with the 38 proteins that were also detected via mass spectrometry (spermatozoa centrosomal proteins) in the shaded regions, sorted by specific centrosome location (Centriolar, PCM, Other location in the Centrosome, and Tubulins). Proteins are displayed with some of their common names, UniProt IDs, and mass spectrometry references, with the percent coverage reported, if available. Staining results in U2OS cells (U2OS staining) are summarized as either “Yes” (indicating that the antibody tested detected signal at the centrosome – as measured by colocalization with γ-tubulin or POC1B-5G5A7 or POC1B-5G5A10), “No” (not detected at the centrosome), or “Not Tested.” Staining results in the sperm (Sperm staining) indicate the location of labeling and quantity relationship (e.g., DC>PC means that staining was more intense in the DC than the PC, and capitulum means that signal was detected in the capitulum area) or “No” (not detected or not enriched in the neck region). The lowest or the best antibody dilution used in immunostaining is shown along with the antibody source and reference. C#, catalog number; * indicates an antibody that had excess non-specific signal or additional staining outside the neck region.

Supplementary References

1 Manandhar, G. & Schatten, G. Centrosome reduction during Rhesus spermiogenesis: gamma-tubulin, centrin, and centriole degeneration. *Molecular reproduction and development* **56**, 502-511, doi:10.1002/1098-2795(200008)56:4<502::AID-MRD8>3.0.CO;2-Q (2000).

2 Baker, M. A. *et al.* Head and flagella subcompartmental proteomic analysis of human spermatozoa. *Proteomics* **13**, 61-74, doi:10.1002/pmic.201200350 (2013).

3 Baker, M. A. *et al.* Identification of gene products present in Triton X-100 soluble and insoluble fractions of human spermatozoa lysates using LC-MS/MS analysis. *Proteomics Clin Appl* **1**, 524-532, doi:10.1002/prca.200601013 (2007).

4 Amaral, A. *et al.* Human sperm tail proteome suggests new endogenous metabolic pathways. *Mol Cell Proteomics* **12**, 330-342, doi:10.1074/mcp.M112.020552 (2013).

5 Wang, G. *et al.* In-depth proteomic analysis of the human sperm reveals complex protein compositions. *Journal of proteomics* **79**, 114-122, doi:10.1016/j.jprot.2012.12.008 (2013).

6 Lin, Y. C. *et al.* Human microcephaly protein CEP135 binds to hSAS-6 and CPAP, and is required for centriole assembly. *EMBO J* **32**, 1141-1154, doi:10.1038/emboj.2013.56 (2013).

7 Pearson, C. G., Osborn, D. P., Giddings, T. H., Jr., Beales, P. L. & Winey, M. Basal body stability and ciliogenesis requires the conserved component Poc1. *J Cell Biol* **187**, 905-920, doi:10.1083/jcb.200908019 (2009).

8 Venoux, M. *et al.* Poc1A and Poc1B act together in human cells to ensure centriole integrity. *J Cell Sci* **126**, 163-175, doi:10.1242/jcs.111203 (2013).

9 Tsang, W. Y. *et al.* Cep76, a centrosomal protein that specifically restrains centriole reduplication. *Dev Cell* **16**, 649-660, doi:10.1016/j.devcel.2009.03.004 (2009).

10 Chang, C. W., Hsu, W. B., Tsai, J. J., Tang, C. J. & Tang, T. K. CEP295 interacts with microtubules and is required for centriole elongation. *J Cell Sci* **129**, 2501-2513, doi:10.1242/jcs.186338 (2016).

11 Zheng, X. *et al.* Conserved TCP domain of Sas-4/CPAP is essential for pericentriolar material tethering during centrosome biogenesis. *Proc Natl Acad Sci U S A* **111**, E354-363, doi:10.1073/pnas.1317535111 (2014).

12 Gromley, A. *et al.* A novel human protein of the maternal centriole is required for the final stages of cytokinesis and entry into S phase. *J Cell Biol* **161**, 535-545, doi:10.1083/jcb.200301105 (2003).

13 Stevens, N. R., Dobbelaere, J., Wainman, A., Gergely, F. & Raff, J. W. Ana3 is a conserved protein required for the structural integrity of centrioles and basal bodies. *J Cell Biol* **187**, 355-363, doi:10.1083/jcb.200905031 (2009).

14 Azimzadeh, J. *et al.* hPOC5 is a centrin-binding protein required for assembly of full-length centrioles. *J Cell Biol* **185**, 101-114, doi:10.1083/jcb.200808082 (2009).

15 Hossain, D., Javadi Esfehani, Y., Das, A. & Tsang, W. Y. Cep78 controls centrosome homeostasis by inhibiting EDD-DYRK2-DDB1VprBP. *EMBO Rep* **18**, 632-644, doi:10.15252/embr.201642377 (2017).

16 Singla, V., Romaguera-Ros, M., Garcia-Verdugo, J. M. & Reiter, J. F. Ofd1, a human disease gene, regulates the length and distal structure of centrioles. *Dev Cell* **18**, 410-424, doi:10.1016/j.devcel.2009.12.022 (2010).

17 Kodani, A. *et al.* Centriolar satellites assemble centrosomal microcephaly proteins to recruit CDK2 and promote centriole duplication. *eLife* **4**, doi:10.7554/eLife.07519 (2015).

18 Comartin, D. *et al.* CEP120 and SPICE1 cooperate with CPAP in centriole elongation. *Curr Biol* **23**, 1360-1366, doi:10.1016/j.cub.2013.06.002 (2013).

19 Leidel, S., Delattre, M., Cerutti, L., Baumer, K. & Gonczy, P. SAS-6 defines a protein family required for centrosome duplication in C. elegans and in human cells. *Nat Cell Biol* **7**, 115-125, doi:10.1038/ncb1220 (2005).

20 Liska, F. *et al.* Rat hd mutation reveals an essential role of centrobin in spermatid head shaping and assembly of the head-tail coupling apparatus. *Biology of reproduction* **81**, 1196-1205, doi:10.1095/biolreprod.109.078980 (2009).

21 Cizmecioglu, O. *et al.* Cep152 acts as a scaffold for recruitment of Plk4 and CPAP to the centrosome. *J Cell Biol* **191**, 731-739, doi:10.1083/jcb.201007107 (2010).

22 Kim, T. S. *et al.* Hierarchical recruitment of Plk4 and regulation of centriole biogenesis by two centrosomal scaffolds, Cep192 and Cep152. *Proc Natl Acad Sci U S A* **110**, E4849-4857, doi:10.1073/pnas.1319656110 (2013).

23 Sonnen, K. F., Gabryjonczyk, A. M., Anselm, E., Stierhof, Y. D. & Nigg, E. A. Human Cep192 and Cep152 cooperate in Plk4 recruitment and centriole duplication. *J Cell Sci* **126**, 3223-3233, doi:10.1242/jcs.129502 (2013).

24 Sir, J. H. *et al.* A primary microcephaly protein complex forms a ring around parental centrioles. *Nat Genet* **43**, 1147-1153, doi:10.1038/ng.971 (2011).

25 Fong, K. W., Choi, Y. K., Rattner, J. B. & Qi, R. Z. CDK5RAP2 is a pericentriolar protein that functions in centrosomal attachment of the gamma-tubulin ring complex. *Mol Biol Cell* **19**, 115-125, doi:10.1091/mbc.E07-04-0371 (2008).

26 O'Rourke, B. P. *et al.* Cep192 controls the balance of centrosome and non-centrosomal microtubules during interphase. *PLoS One* **9**, e101001, doi:10.1371/journal.pone.0101001 (2014).

27 Schmidt, K. N. *et al.* Cep164 mediates vesicular docking to the mother centriole during early steps of ciliogenesis. *J Cell Biol* **199**, 1083-1101, doi:10.1083/jcb.201202126 (2012).

28 Lee, J. E. *et al.* CEP41 is mutated in Joubert syndrome and is required for tubulin glutamylation at the cilium. *Nat Genet* **44**, 193-199, doi:10.1038/ng.1078 (2012).

29 Klinger, M. *et al.* The novel centriolar satellite protein SSX2IP targets Cep290 to the ciliary transition zone. *Mol Biol Cell* **25**, 495-507, doi:10.1091/mbc.E13-09-0526 (2014).

30 Sillibourne, J. E. *et al.* Primary ciliogenesis requires the distal appendage component Cep123. *Biology open* **2**, 535-545, doi:10.1242/bio.20134457 (2013).

31 Shao, X., Tarnasky, H. A., Lee, J. P., Oko, R. & van der Hoorn, F. A. Spag4, a novel sperm protein, binds outer dense-fiber protein Odf1 and localizes to microtubules of manchette and axoneme. *Dev Biol* **211**, 109-123, doi:10.1006/dbio.1999.9297 (1999).

32 Yuan, S. *et al.* Spata6 is required for normal assembly of the sperm connecting piece and tight head-tail conjunction. *Proc Natl Acad Sci U S A* **112**, E430-439, doi:10.1073/pnas.1424648112 (2015).
